# Self-regulated learning strategies adopted by successful Chinese nursing students in the process of learning Nursing English

**DOI:** 10.1371/journal.pone.0308353

**Published:** 2024-08-08

**Authors:** Lina Wang, Xuesong (Andy) Gao, Fan Zhang, Fang Sun, Guangsheng Wan

**Affiliations:** 1 Foreign Languages Faculty, Shanghai University of Medicine and Health Sciences, Shanghai, China; 2 School of Education, University of New South Wales, Sydney, Australia; 3 School of Nursing and Health Management, Shanghai University of Medicine and Health Sciences, Shanghai, China; Golestan University, ISLAMIC REPUBLIC OF IRAN

## Abstract

As the number of foreign patients and the frequency of international academic exchanges increase, English proficiency has become increasingly essential for Chinese nurses in the treatment and nursing processes, clinical academic exchanges, and ongoing education. However, the overall English proficiency of Chinese nurses is generally inadequate, greatly depending on the English that they acquire during their nursing education. This study aims to explore the challenges encountered by Chinese EFL (English as a Foreign Language) nursing students in the process of learning Nursing English, along with the effective self-regulated learning strategies they adopt to overcome these challenges. Data were collected from nine Chinese EFL nursing students through their reflective journals and thematic analysis was applied. Data analysis revealed the variety of challenges EFL nursing students encountered, including language-related challenges, which are linguistic difficulties that relate to Nursing English learning itself, such as Nursing English vocabulary and terminology, English-to-English translation, limited listening comprehension, and the gap between textbook knowledge and its practical application; learner-related challenges, which are difficulties that affect students’ emotional, affective, and mental state, primarily caused by uncertainty about the significance of Nursing English, the unexpected difficulty of Nursing English, and failing quizzes; and context-related challenges, which are difficulties relate to social, cultural, and educational context, such as insufficient learning resources, a lack of language environment, and peer pressure. To surmount these challenges, the participants adopted diverse self-regulated learning strategies, including setting goals, previewing in advance and reviewing in time, utilizing word roots, prefixes, and suffixes to facilitate vocabulary learning, repeating, practicing with sounds and writing systems, translating, highlighting and using imagery to overcome language-related challenges; believing in the usefulness and significance of Nursing English, keeping a growth mindset, enjoying Nursing English learning and teacher support and maintaining grit in learning Nursing English to overcome learner-related challenges; and integrating resources, creating supportive language environments and seeking assistance from teachers and cooperating with peers to overcome context-related challenges. Based on these findings, implications are drawn for Nursing English teachers, material designers, curriculum developers, and program designers. We suggest incorporating explicit strategy instruction into regular Nursing English education to enhance nursing students’ self-regulated learning.

## Introduction

According to the *China Health Statistical Yearbook 2022*, there were 5,019,422 registered nurses in China by the end of 2021 [1]. The number of foreign patients admitted by hospitals and the frequency of academic exchanges with other countries are both on the rise as a result of the rapid development of China’s social economy and the rising frequency of exchanges with other countries. Thus, English proficiency is increasingly essential for nurses in the treatment and nursing processes, clinical academic exchanges, and their ongoing education. Medical institutions, especially 3A hospitals (the top level of hospital ranking in China) and those with foreign investments, have recognized the importance of English proficiency at all levels of nursing. However, the status quo in China shows that nurses’ overall English proficiency is inadequate, which negatively impacts the quality and effectiveness of foreign-related nursing tasks and hampers nurses’ opportunities for continued education [2].

The English proficiency of Chinese nurses greatly depends on the language skills they acquire in nursing schools. Fluent communication in a patient’s preferred language can significantly enhance patient satisfaction and improve healthcare outcomes [3]. However, nursing staff in China often exhibit a deficiency in English proficiency, a shortfall that notably impacts the quality and efficiency of foreign-related nursing work [2]. Nursing English is a specialized branch within the broader field of English for Specific Purposes (ESP). It specially addresses how nurses (as opposed to doctors and other healthcare professionals and paraprofessionals) use English, in both clinical settings and educational contexts [4]. As a compulsory course in nursing education in China, Nursing English plays a vital role in fostering the English proficiency of nursing students [5]. Proficiency in Nursing English could boost students’ success in nursing programs and their professional careers [6]. The Nursing English competence is increasingly relevant in the competitive global job market after graduation and when applying for further education programs. However, competence represents only the outcome, not the experiential process. Thus, it is important to adopt a comprehensive perspective that takes into account both the challenges nursing students encounter in the process of learning Nursing English and the corresponding self-regulated learning strategies they adopt to overcome these challenges.

Self-regulated learning strategies are actions and processes directed at acquiring information or skill. According to social cognitive theory, self-regulated learning is not determined merely by personal processes, these processes are assumed to be influence by environmental and behavioral events in reciprocal fashion [7]. These strategies play a crucial role in fostering students’ self-regulated learning capacity, which in turn improves their language proficiency. Whether they can effectively employ various self-regulated learning strategies to stimulate, maintain and adjust their cognition, emotions and behaviors is a crucial determinant in achieving their learning objectives [8].

Existing studies on self-regulated learning strategies have primarily focused on general English learning [9–12] rather than on ESP learning. In recent years, however, research has started to explore the unique challenges encountered by nursing students in learning Nursing English, as well as effective strategies to promote learning in this specialized field. Most existing research has focused on ESL (English as a Second Language) nursing students [13–17] and EAL (English as an Additional Language) nursing students [18–20], with relatively little attention given to EFL nursing students. As a predominantly EFL country, China possesses a distinctive educational and sociocultural background. Consequently, Chinese nursing students may encounter distinct challenges compared to their ESL or EAL counterparts in learning Nursing English. Furthermore, the strategies discussed in existing studies are primarily teaching or learning strategies proposed by nursing teachers or researchers rather than those developed from the perspective of the learners themselves. To the best of our knowledge, no study has thoroughly examined the self-regulated learning strategies adopted by successful EFL nursing students in learning Nursing English. Successful learners are characterized by their ability to employ more effective and efficient learning strategies for accessing and utilizing knowledge. They are self-motivated, and they monitor and adjust their behaviors when learning does not occur [21]. Understanding the experiences of successful English learners and exploring the learning strategies that can be emulated by others holds both theoretical and practical significance [22]. The current study explores the challenges encountered by nine successful Chinese EFL nursing students in their process of learning Nursing English. We are particularly interested in the self-regulated learning strategies these students have adopted to overcome these challenges. To the best of our knowledge, this is the first qualitative study to explore the self-regulated learning strategies adopted by successful learners in an EFL context specially within Nursing English. The insights gained from this research could significantly enhance our understanding of how EFL nursing students effectively adopt self-regulated learning strategies. The findings are expected to have important implications for Nursing English educators, materials designers, curriculum developers, program designers, and all others involved in nursing education.

## Methodology

This study employed a qualitative research design to explore the challenges encountered by Chinese EFL nursing students and the self-regulated learning strategies they adopted in response to these challenges during their Nursing English learning process. To do so, the following research questions were formulated:

RQ1: What challenges did Chinese EFL nursing students encounter in their learning of Nursing English?

RQ2: What self-regulated learning strategies did Chinese EFL nursing students adopt in their learning of Nursing English?

### Research context

Given the global shortage of nurses, the employment pressure on nursing students in China is significantly lower than that faced by graduates from other majors. Furthermore, nursing is considered one of the more lucrative career options available [23], contributing to its popularity as a major in China. Annually, a substantial number of nursing graduates in China is engaged in foreign-related nursing work. Although English is not an official language in China, it is a compulsory subject in elementary, secondary, and tertiary education. Moreover, Nursing English is a mandatory course for nursing majors at higher educational levels.

The WorldSkills Competition, a biennial world championship of vocational skills, hosted in different regions around the world. This event aims to highlight the benefits of learning vocational skills and to promote ‘parity of esteem’ between vocational and academic qualifications. China is among the 85 member countries and regions of WorldSkills, actively participating in organizing national skills competitions aid in selecting contestants, thereby preparing the current workforce and talent for future positions. As of 2022, there are competitions in 61 skill areas across five trades, with health and social care recognized as one of the skill areas within the social and personal services trade.

### Participants

To uncover, understand, and gain insights into how successful Nursing English learners strategically self-regulate their learning process, we employed a purposive and criterion-based sampling approach to recruit participants [24]. The recruitment of participants was based on the following criteria: 1) participants must be current students or graduates of nursing programs at the target university, and 2) they must have participated in and won awards in either WorldSkills Competitions or national/municipal competitions that supported the selection of contestants for WorldSkills Competitions. Following the approval from the Human Research Ethics Committee of the School of Nursing and Health Management at Shanghai University of Medicine and Health Sciences, we identified competition winners at the target university. From February 10, 2022 to March 30, 2022, we reached out to these individuals via WeChat, a widely used social media platform in China, and asked for their participation. Only winners who provided written informed consent were included in the study and they were free to withdraw at any time.

Nine participants who met these criteria were eventually recruited. They expressed a willingness to share their experiences with learning Nursing English and voluntarily agreed to participate in the study. The average age of these participants was approximately 23.33 years, and they had been learning Nursing English for an average duration of about 3.55 years. The group consisted of one male and eight female participants, who came from various nursing programs, and represented different levels of nursing education in China. In order to maintain anonymity, pseudonyms have been used throughout this study r (see Table 1).

**Table 1:** Participant profiles.

Pseudonym	Age	Gender	Nursing Program	Duration of Nursing English study (years)
Chen	21	Female	Diploma of Nursing	2
Lin	25	Female	Bachelor of Nursing	5
Liu	21	Female	Bachelor of Nursing	4
Shan	22	Female	Diploma of Nursing	7
Tang	24	Female	Diploma of Nursing	3
Wang	24	Male	Master of Nursing	2
Wu	22	Female	Bachelor of Nursing	2
Yang	24	Female	Bachelor of Nursing	4
Zhu	27	Female	Diploma of Nursing	3

### Data collection

After all participants had signed the written informed consent forms, we initiated the data collection process by inviting them to write reflective journals. Reflective journals are recognized as powerful research tools for capturing students’ reflective practice, conceptual changes, thinking, and learning processes [25]. Journaling encourages participants to identify challenges in their learning and describe how they achieved a solution [26]. To guide their reflections, we presented ‘A Long Way of Learning’ by Runqing Liu [27], a renowned foreign language learner and educator in China. Their reflections were prompted by the following four questions: 1) What challenges have you encountered in learning Nursing English? 2) What factors do you think have influenced your learning of Nursing English? 3) Can you recall any critical events that affected your learning of Nursing English (such as courses, participating in competitions, hospital internships, etc.)? Please describe these events in detail. 4) What learning strategies have you adopted to self-regulate your learning of Nursing English? Ultimately, we collected nine reflective journals, totaling 21,339 Chinese characters.

### Data analysis

The data from reflective journals were manually coded and analyzed through a cyclical and evolving process of coding and recoding, employing thematic analytical approaches [28]. In the first cycle, we read the data line by line, marked relevant data segments in different colors, and recorded comments on the data that struck the researchers as interesting in the transcript margins. In the second cycle, we reread the data, marked additional segments, and recorded further comments. We then coded the marked data into basic-level concepts, such as ‘felt confused about the significance of learning Nursing English’ and ‘speaking English became enjoyable’. In the third cycle, we categorized similar basic-level concepts. For instance, the concepts ‘I felt anxious when I tried to recite this terminology’ and ‘I failed the class quiz and freaked out’ were grouped into the analytical category of ‘learner-related challenges. Corresponding self-regulated learning strategies were grouped into themes, such as ‘we should always believe that as long as we work hard, we can succeed in the end’ and ‘I do believe that I will strive to break through gradually in the future through further study and accumulation of knowledge and experience’ being grouped into the theme ‘keep a growth mindset’.

## Findings

The data analysis identified three categories of challenges that Chinese EFL nursing students encountered in their learning of Nursing English: language-related, learner-related, and context-related challenges. As shown in Table 2, language-related challenges were reported most frequently (54.5%), followed by learner-related (27.3%) and context-related (18.2%) challenges. Furthermore, the participants adopted a variety of self-regulated learning strategies to overcome these challenges.

**Table 2:** Three categories of challenges and relevant self-regulated learning strategies.

Category of the challenge	Frequency & percentage	Self-regulated learning strategies
**Language-related**	48 (54.5%)	
Linguistic difficulties that relate to Nursing English learning itself, such as Nursing English vocabulary and terminology, English-to-English translation, limited listening comprehension, and the gap between the textbook knowledge and its practical application		Set goals Preview in advance and review in time Utilize word roots, prefixes, and suffixes to facilitate vocabulary learning Repeat, practice with sounds and writing systems, translate, and highlight Use imagery
**Learner-related**	24 (27.3%)	
Difficulties that affect students’ emotional, affective, and mental state, primarily caused by uncertainty about the significance of Nursing English, the unexpected difficulty of Nursing English, and failing quizzes		Believe in the usefulness and significance of Nursing English Keep a growth mindset Enjoy Nursing English learning and teacher support Maintain grit in learning Nursing English
**Context-related**	16 (18.2%)	
Difficulties relate to social, cultural, and educational context, such as insufficient learning resources, a lack of language environment, and peer pressure		Integrate resources Create supportive language environments Seek assistance from teachers and cooperate with peers

### Challenges encountered by Chinese EFL nursing students in their Nursing English learning process

#### Language-related challenges

Language-related challenges refer to linguistic difficulties relate to Nursing English learning itself, such as Nursing English vocabulary and terminology, English-to-English translation, limited listening comprehension, and the gap between textbook knowledge and its practical application.

The most significant language-related challenge reported by participants was, unsurprisingly, the complexity of Nursing English vocabulary and terminology. Participants used words such as ‘complicated’, ‘lengthy’, and ‘obscure’ to describe Nursing English vocabulary and terminology. For instance, Lin noted:

I found that the Nursing English words were very complicated. For me, reciting a new Nursing English word was like remembering a lot of unintelligible codes (Lin).

The obscure and lengthy Nursing English vocabulary was extremely difficult and challenging for me (Shan).

Another language-related challenge involved English-to-English translation where learners need to not only understand the specialized vocabulary but also translate it into simpler English that can be easily understood by patients. Lin shared her experience during the WorldSkills Competition:

In the WorldSkills Competition, we needed to explain medical terms to patients in simple English, necessitating English-to-English translation. Expressing these professional terms briefly in English was also a challenge (Lin).

During the training for the same competition, Yang encountered similar difficulties:

My coach and I frequently struggled to figure out how to describe disease symptoms in clear and simple English (Yang).

A third language-related challenge was limited listening comprehension. Tang expressed difficulty in understanding her native English teachers:

In a non-English environment, my listening ability was not adequate to fully understand the speakers’ key points… This s likely a common problem for most Chinese English learners (Zhu).

Finally, participants noted the gap between the Nursing English learned from textbooks and its practical application. Lin recalled her training for the WorldSkills Competition and her clinical practice experiences:

In training for the WorldSkills Competition… I frequently spoke Chinglish… Compared to the clinical practice of Nursing English at United Family Healthcare, the Nursing English I learned from textbooks was not very practical (Lin).

#### Learner-related challenges

Learner-related challenges refer to the difficulties that affect nursing students’ emotional, affective, and mental states. These challenges are primarily driven by uncertainty about the significance of Nursing English, the unexpected difficulty of the subject, and failing quizzes.

Most participants expressed psychological challenges, frequently using terms such as ‘pressure’, ‘shock’, ‘self-doubt’, ‘anxiety’, ‘confusion’, ‘tiresome’, and ‘stress’ to describe their emotional states during the process of learning Nursing English. One primary cause of these challenges was uncertainty about the significance of Nursing English.

Despite English being a foreign language in China, and rarely used outside specific contexts, even in Shanghai, an international metropolis like Shanghai, students seldom engage in immersive English-language environments. This limited exposure led some participants to question the significance of learning Nursing English. Chen articulated this sentiment:

Many of us felt confused about the significance of learning Nursing English. After all, we are Chinese. Why should we learn Nursing English if we are not planning to study abroad (Chen)?

English is a compulsory subject throughout elementary and secondary education in China, with many students beginning their English studies in elementary school or even kindergarten. However, ESP, which is typically introduced at the tertiary level, differs significantly from general English learning. ESP is distinguished by introducing many complicated terminology systems and an extensive vocabulary, which makes listening, speaking, reading, and writing in ESP particularly challenging. Liu described the daunting nature of these initial encounters with Nursing English:

At the beginning of learning Nursing English, I found it extremely difficult. Medical terms are usually unfamiliar, exceedingly long and challenging to pronounce. While I recall their meanings when reviewing them in the textbook, they seem like entirely new words once the book was closed. This experience was common among my classmates (Liu).

The psychological impact of this complexity was profound, as Wu explained:

In learning Nursing English, the intimidating terminology, enormous vocabulary, and the overall difficulty in listening, speaking, reading, and writing made me anxious, especially when trying to memorize terminology (Wu).

A further example is Wu’s overwhelming experience and her feeling lost when she prepared for the WorldSkills Competition:

I was shocked when the written test paper for the first round of selection [for the WorldSkills Competition] was placed in front of me. It was filled with multiple-choice questions in English and a multitude of professional medical terms. The pressure was immense,…and despite all my preliminary preparations, I still felt overwhelmed (Wu).

Failing quizzes further exacerbated these psychological challenges. Shan shared her distress following a failed quiz:

I freaked out because I failed the quiz. I was certain that I had carefully studied the new words. Why was my score still so low (Shan)?

### Context-related challenges

Context-related challenges compass difficulties arising from social, cultural, and educational contexts, such as insufficient learning resources, a lack of language environment, and peer pressure.

In China, the availability of Nursing English textbooks and learning materials is severely limited. This scarcity of resources has significantly hindered the learning of Nursing English among Chinese nursing students. As described by the participants described it:

In Nursing English learning, some external factors, such as the lack of authoritative learning resources, have impacted our effective learning (Liu).

There were very few textbooks and learning resources related to Nursing English available in China (Lin).

The absence of an immersive English-language environment is another context-related challenge. As English is not an official language in China, Chinese EFL learners find their opportunities to use English outside the classroom severely limited. This issue is particularly acute for learners of Nursing English:

The absence of a specific English communication environment led me to develop the bad habit of ‘dumb English’ (Tang).

In China, the medical environment where English was spoken consisted mainly of foreign-funded hospitals and VIP clinics of hospitals (Liu).

Peer pressure also presents a significant context-related challenge. The behavior of peers can affect the learning of Nursing English, either directly or indirectly. Shan considered dropping out of the Nursing English class due to peer pressure:

After the first class, a few students sneaked out the back door…After several classes, our Nursing English class had dwindled to one teacher and ten students…By the time we got to the word root ‘hyper’, only four or five students remained. My classmates’ departure made me waver on the verge of skipping class, and I was ready to give up (Shan).

### Self-regulated learning strategies to overcome challenges with learning Nursing English

Previous studies have indicated that how well language learners manage challenges in their learning process largely depends on their self-regulated learning abilities [10, 29]. The effective employment of self-regulated learning strategies is critical for successful self-regulated learning. All participants in this study encountered various language-related, learner-related, and context-related challenges and employed a range of self-regulated learning strategies to overcome these challenges. These strategies have enabled them to overcome the challenges they faced in learning Nursing English.

### Self-regulated learning strategies to overcome language-related challenges

The self-regulated learning strategies adopted by participants to address language-related challenges include setting goals, previewing in advance and reviewing in time, utilizing word roots, prefixes, and suffixes to facilitate Nursing English vocabulary learning, repeating, practicing with sounds and writing systems, translating, highlighting, and using imagery.

### Set goals

Goal setting is the process of establishing clear and usable targets or objectives for learning [30]. Previous studies have indicated that goal setting affects students’ performance and enhances achievement in language learning [31]. The participants in this study actively managed their Nursing English studies by setting goals. Moreover, based on the goals they had set for themselves, they organized their learning through self-monitoring and self-evaluation:

During the first few weeks of school, I decided to catch up and immediately set learning goals to keep up with the course (Tang).

Having a clear goal motivated me more (Shan).

I set a small goal–to recite ten words each day. This lasted for six days…I reflected on my learning progress…and realized I had learned too much without proper assimilation. Moreover, my goal was too ambitious (Shan).

### Preview in advance and review in time

By previewing the Nursing English contents that they were about to study, learners could activate their previous knowledge and build background knowledge, which benefited their understanding and digestion of the content presented in class. After class, they promptly reviewed the learning content to reinforce important and difficult points.

It was necessary to translate and preview the professional terms throughout the textbook because there were so many of them. Many of my textbooks were annotated in Chinese (Chen).

The most important learning activity for me was reviewing the Nursing English content of the day bit by bit after class every day…I reviewed the teachers’ slides several times and repeatedly wrote down the content until I could roughly summarize the lesson for the day (Wang).

I would preview the content that would be taught the next day, comprehending the main idea, and grasping the theme. I looked up unfamiliar medical terms and new words in advance and annotated them to strengthen my understanding, which also deepened my impression of the words. After studying a chapter, I made a learning outline, reviewed the key points of each chapter, and used a mind map to summarize what I had learned (Tang).

Our participants considered Nursing English vocabulary and terminology very difficult. Learning and memorizing this vocabulary and terminology was perceived as the primary language-related challenge. They adopted several self-regulated learning strategies to cope with the difficulty of retaining this information.

### Utilize word roots, prefixes, and suffixes to facilitate vocabulary learning

The participants utilized word roots, prefixes, and suffixes and summarized word formation rules to facilitate their learning of Nursing English vocabulary. For example:

While learning Nursing English, I gradually learned the word formation rules. I used prefixes and suffixes to distinguish Nursing English words (Lin).

I realized that medical vocabulary follows specific word formation rules when foreign teachers explained the roots, prefixes, and suffixes. By dividing and reformatting each part, I could learn even the most complex and lengthy medical terminology (Wu).

We learned vocabulary and grammar through rote learning. However, we must still adhere to certain scientific principles. For instance, prefixes, suffixes, and roots often represent the most fundamental meanings of words (Liu).

### Repeat, practice with sounds and writing systems, translate, and highlight

The participants adopted strategies such as repeating, practicing with sounds and writing systems, translating, and highlighting. Repetition is widely used among Chinese EFL learners. Using self-made flashcards and modern technology, such as dubbing apps, participants repeatedly practiced the newly-learned Nursing English content with sounds and writing systems. They also paid close attention to intonation and emotion when dubbing. This emphasis was important because most participants appreciated the significance of humanistic care in nursing, and applying appropriate intonation and emotion could promote communication between patients and nurses. They also focused on translating and highlighting difficult points:

I repeatedly dictated difficult words each day to deepen my impression…I began to look up common diagnostic words and nursing operation words, using dictionaries or Google to find authoritative explanations. For nursing vocabulary, I would first explain them in Chinese and then translate them into simple English (Wu).

I dubbed my favorite films and television shows using English dubbing apps (Yang).

I used flashcards to aid my memorization of new words (Liu).

I recommended that my classmates use flashcards to improve recitation. Initially, new words were sorted by roots and affixes, and related words were written on the same card. Before breakfast, I reviewed the vocabulary using the flashcards that I had brought along (Tang).

I marked the phonetic symbols and the corresponding Chinese translation in the margins of the article…In addition, I framed some nursing-specific phrases and frequently-used dialogues within the text’ s sample dialogue sections with colored pencils (Zhu).

### Use imagery

Imagery was also commonly used among the participants in this study. According to dual coding theory, information is better comprehended and remembered when stored in two codes (such as a verbal system for language and a nonverbal system for imagery) than in one code alone. Participants in this study employed visuals, such as the Illustrated Guide to Medical Terminology, to enhance their Nursing English learning. The content was encoded verbally and nonverbally during this procedure. As a result, the information was elaborated upon, potentially promoting increased comprehension and a strengthened memory trace [32].

I used reference materials like the Illustrated Guide to Medical Terminology to assist word memorization with the help of pictures (Wu).

Instead of translating the word ‘book’ into Chinese, you should immediately conjure up an image of a book. Your progress in learning Nursing English terms will be fast if your imagination is engaged (Wang).

### Self-regulated learning strategies to overcome learner-related challenges

Although the participants in this study experienced self-doubt, stress, confusion, and other challenging emotions when they started learning Nursing English, it was important that they made adjustments and adopted appropriate self-regulated learning strategies for their psychological development. The self-regulated learning strategies that our participants adopted to overcome learner-related challenges included believing in the usefulness and significance of Nursing English, keeping a growth mindset, enjoying Nursing English learning and teacher support and maintaining grit in learning Nursing English.

### Believe in the usefulness and significance of Nursing English

In China, a typical EFL country, opportunities for Chinese EFL learners to use English are relatively scarce. Given this social and educational background, some people believe that learning English is unnecessary and advocate for the removal of English from China’s college entrance examination. Yang initially shared this view, considering local dialects more important for nurses in China than English. However, she changed her mind after her internships in several foreign-funded hospitals. Furthermore, most participants in this study recognized the usefulness and significance of Nursing English for their academic learning and future professional development. This belief significantly enhanced their motivation to thoroughly learn Nursing English.

The local dialect seemed to be more important than English when communicating with patients…But the internship opportunities in foreign-funded hospitals made me realize that having excellent English skills could increase career opportunities for nursing practitioners (Yang).

If you want to progress in nursing, you must keep updated with both domestic and international nursing trends…This requires you a good command of Nursing English (Liu).

### Keep a growth mindset

Language mindset refers to an individual’s perception of their own language learning ability [33]. Dweck divided mindset into two categories: 1) a fixed mindset is the belief that one’s basic qualities and attributes (e.g. intelligence, aptitude, personality, and morality) are innate and, therefore, cannot be changed; while 2) a growth mindset is the beliefs that one’s qualities can be cultivated over time through hard work and strategic actions [34]. Language mindset is crucial because it has implications for how people respond to adverse situations [35]. Learners with a growth mindset consider challenges as opportunities for growth, set realistic learning objectives, and do their best to achieve those objectives [36]. Participants in this study maintained a growth mindset, firmly believing in their ability to overcome adverse situations and succeed in learning Nursing English through hard work.

We should always believe that as long as we work hard, we can succeed…There will definitely be a turning point if we persistently work hard and gradually (Wang).

I did believe that I would gradually make breakthroughs in the future through further study and the accumulation of knowledge and experience (Liu).

### Enjoy Nursing English learning and teacher support

As indicated by the broaden-and-build theory, learners in an enjoyable language learning environment are more likely to be motivated to seek opportunities to learn the target language. Foreign language enjoyment is a complex emotion, capturing interacting dimensions of challenge and perceived ability that reflect the human drive for success in the face of difficult tasks [37]. It can boost learners’ capacity to notice things in the classroom environment and strengthen their awareness of language input, increasing the probability of their absorbing more of the foreign language [38]. The participants in this study enjoyed both learning Nursing English and teacher support:

I have made significant progress in a short period. This progress naturally led to an increase in confidence in conversation, and speaking English became enjoyable for me (Yang).

At the beginning, I started learning Nursing English to emulate the proficient learners. Gradually, I became the proficient learner among my peers…I could feel the enjoyment of learning Nursing English (Shan).

The foreign teachers encouraged us to speak English…. What touched me was that they would listen patiently without interrupting us, regardless of our English level. They would help us refine our answers when we encountered difficulties. When I heard their comment ‘good job’ again and again, my confidence grew, and I became more willing to take the initiative independently to face new challenges…Fortunately, when I encountered problems in my study, I could ask the foreign teachers for help anytime. They warmly invited me to join them for dinner, providing more opportunities to practice my speaking English. As my friendship with the teachers grew, I naturally became less shy about speaking English (Tang).

In preparing for the competition, I could not have succeeded without the help of Nursing English teachers. Their support also strengthened my motivation and confidence to continue learning Nursing English (Liu).

Although American teachers also focused on teaching grammar, they did not overly emphasize it. This approach helped to create a relaxing atmosphere, enhancing our enjoyment of learning. Moreover, some classroom practices of American teachers, such as not requiring students to stand up to answer questions and giving small gifts as incentives, narrowed the distance between students and teachers. They always remembered our birthdays which also promoted a good teacher–student relationship and facilitated the teaching and learning of Nursing English (Wang).

### Maintain grit in learning Nursing English

Grit refers to perseverance and passion for long-term goals [39]. Learning a foreign language in the classroom context is an arduous endeavor that requires grit, especially when there is little input from that language in one’s natural environment [40]. Learning Nursing English is a long process, and learners are bound to encounter challenges, difficulties, setbacks, and discouragements. Gritty students don not give up in discouraging setbacks and downturns [41]. The participants in this study demonstrated grit and maintained it in their learning of Nursing English. They took the first step and persevered, consistently attending classes despite some peers dropping out.

I insisted on attending Nursing English classes from Level 1 to Level 4…Within two years, my vocabulary expanded to 7,000 words, and I advanced from a beginner to an advanced Nursing English learner (Shan).

The key was to take the first step learning Nursing English and stick with it (Liu).

Learning Nursing English demanded perseverance…Without patience, we would likely give up halfway (Chen).

Learning Nursing English was like fighting a protracted war (Chen).

### Self-regulated learning strategies to overcome context-related challenges

Our participants adopted several self-regulated learning strategies to overcome context-related challenges, such as integrating resources, creating supportive language environments and seeking assistance from teachers and cooperating with peers.

### Integrate resources

Chinese EFL learners are increasingly turning to the internet as a resource for learning Nursing English. The convenience it offers in accessing and obtaining an endless supply of authentic materials in target languages makes the internet a resource that enriches and expands language learning [42]. In order to overcome the challenge of insufficient resources, the participants in this study actively integrated resources on the internet to facilitate their Nursing English learning. For example, in order to describe a disease in idiomatic English, they referred to doctors’ descriptions of patients’ conditions in American medical TV dramas online. Their understanding of diseases was furthered by the graphic animated demonstrations in these dramas. They also studied videos of American registered nurses on self–media platforms to learn international practical nurse–patient and doctor–nurse communication skills. They played medical puzzle games as a method to learn medical content, which also helped in overcoming the challenge of the gap between the textbook knowledge and its practical application.

In some case analyses in American TV dramas, the doctors’ descriptions of their patients’ conditions were natural and accurate, and there were even vivid animated demonstrations. We could completely reproduce their description and improvise in our training (Yang).

In order to learn more about the communication styles of some particular medical institutions in the competition, I would consult foreign website resources, such as subscribing to videos of registered nurses in the United States on YouTube. In this way, I learned international practical nurse–patient and doctor–nurse communication skills (Wu).

I would shadow read my favorite passages aloud when I watched American TV series. Because of my major, I watched more TV shows related to healthcare, such as ‘Grey’s Anatomy’ and ‘House M.D.’…At the same time, teachers on YouTube would illustrate and explain the various organizational structures of the human body…The medical puzzle game developed by the University of Hong Kong made us memorize medical terms subconsciously while doing brain teasers (Shan).

### Create supportive language environments

Chinese learners of English have extremely limited opportunities to practice and use Nursing English because China is an EFL country, a setting unfavorable for the process of learning Nursing English. However, our participants took the initiative to create more supportive language environments and made the best use of the available resources. For example, they attended Massive Open Online Courses (MOOCs), watched American medical TV dramas or shows, and made friends with English-speaking expatriates in China.

We should create an English environment to put ourselves in an excellent English learning environment…make friends with foreign teachers, engage in casual conversation, integrate language learning into daily life, watch American TV dramas, listen to English songs, and imagine yourself as a foreigner (Wang).

In my opinion, the Nursing English learning environment was crucial…For example, I would watch medical TV dramas of my interests, such as ‘Grey’s Anatomy’ and ‘The Good Doctor’. I created a Nursing English learning environment in this way (Wu).

### Seek assistance from teachers and cooperate with peers

A neo-Vygotskian perspective views learning as occurring in interaction with more expert others [43]. Through active interaction with teachers and peers, the participants in this study acquired knowledge and skills, such as authentic English expressions and new modes of nurse–patient and therapeutic communication. At the same time, they also cultivated a deeper interest in learning Nursing English.

Later, I began consulting with foreign teachers and English teachers at our school…Finally, the practical application of Nursing English was carried out with teachers or peers for situational simulation (Wu).

Under the guidance of my nursing teacher and my Nursing English teacher, I competed in the competition on behalf of my school and won second prize in the Shanghai Selection Contest of the ‘Health and Social Care program’ of the 46th WorldSkills Competition…Two years later, I teamed up with my junior schoolmate from the Sino–US nursing program. We participated in the 2021 Nursing Skills Competition for Domestic Medical Colleges (Liu).

In the day-after-day training for nursing competitions, my former opponents and I gradually became friends. We discussed the optimization of communication methods, practiced, and reflected together. Standing on the world stage, I interacted with and learnt from nursing practitioners from other countries, and English became a tool for me to connect seamlessly with the rest of the world (Yang).

## Discussion

This study examined the challenges encountered by nine successful Chinese EFL nursing students and the self-regulated learning strategies they adopted in the process of learning Nursing English. Concerning the first research question, the findings revealed that these students encountered language-related, learner-related, and context-related challenges. While previous studies have mainly focused on language-related challenges [15, 44], this study additionally delved into learner- and context-related challenges. Identifying the learner- and context-related challenges revealed hidden internal factors inhibiting their learning of Nursing English. Moreover, some challenges highlighted in this study are uniquely shaped by the specific social and educational contexts in China, such as the context-related challenges of insufficient learning resources and a lack of language environment.

Regarding the second research question, the findings showed that the participants adopted diverse self-regulated learning strategies to surmount these challenges. To tackle language-related challenges, they engaged in setting goals, previewing in advance and reviewing in time, utilizing word roots, prefixes, and suffixes to facilitate Nursing English vocabulary learning, repeating, practicing with sounds and writing systems, translating, highlighting and using imagery. According to social cognitive theory, this is how they effectively adopt self-regulated learning strategies to achieve behavioral self-regulation. To address learner-related challenges, the participants adopted strategies such as believing in the usefulness and significance of Nursing English, keeping a growth mindset, enjoying Nursing English learning and teacher support and maintaining grit in learning Nursing English. According to social cognitive theory, this is how they effectively employ self-regulated learning strategies to achieve covert self-regulation. Lastly, to overcome context-related challenges, they focused on integrating resources, creating supportive language environments and seeking assistance from teachers and cooperating with peers. According to social cognitive theory, this is how they effectively use self-regulated learning strategies to achieve environmental self-regulation.

The successful nursing students acknowledged the significance of goal setting. Notably, this recognition is often absent among less successful learners. Studies have shown that setting specific learning goals can substantially enhance language learning achievement, yet this important learning strategy has been largely ignored in classrooms [30].

In this study, other self-regulated learning strategies were also adopted by successful nursing students in order to overcome language-related challenges. They previewed and promptly reviewed. They repeated the learning content, formally practicing with sounds and writing systems, translating, and highlighting. Yang also found that nursing pre-professionals in Taiwan generally prefer to using both written and verbal repetition when learning medical terminology [45]. For mastering medical vocabulary and terminology, students utilized word roots, prefixes, and suffixes, summarizing rules of word formation. This approach facilitated the understanding and memorization of vocabulary and terminology. This is especially true for learning Nursing English, which has many words with Latin and Greek roots. This strategy is highly effective since each root word can yield a dozen or more English derivatives. By properly utilizing this system of word roots, prefixes, and suffixes, learners can quickly master thousands of words. Moreover, this strategy, unlike rote learning, facilitates meaningful learning of Nursing English vocabulary and ensures the information is stored in long-term memory, simplifying vocabulary retrieval. The participants also used imagery to facilitate their Nursing English learning. Learning vocabulary and professional content in Nursing English through both verbal and imagery modes proved to be more effective in helping learners comprehend and memorize content more effectively. The participants believed in the usefulness and significance of Nursing English. Even though Nursing English may not seem immediately useful in China, where English is not an official language, the participants adjusted their beliefs, specifically after their personal internships in foreign-funded hospitals. Motivated by these experiences, they invested more efforts into their Nursing English learning, believing in its importance for their career development.

In addition, the participants kept a growth mindset regarding their learning of Nursing English, believing that through hard work and strategic actions, they could achieve success. Cultivating a growth mindset is a promising way to sustain self-regulation that has been shown to affect behaviour by partly altering effort attributions [46]. Notably, a growth mindset can be cultivated through interventions. Studies have shown that a brief mindset intervention that informs learners about the potential for intelligence to grow with effort and effective learning strategies can promote lasting academic achievements [47–49].

Moreover, the participants enjoyed learning Nursing English. Indeed, it is essential for learners to enjoy their study of Nursing English rather than just valuing its instrumental benefits, because enjoyment pushed them into action, kept them engaged in learning and maximized their motivation despite difficulties [37, 50]. They also enjoyed having the support of their Nursing English teachers.

The participants in this study also maintained grit in their learning of Nursing English. Grit was positively related with students’ engagement [50]. Chinese EFL nursing students are bound to encounter innumerable challenges and difficulties in the learning of Nursing English, and it is their grit that enables them to stay engaged and ultimately succeed.

The participants turned to the internet to integrate resources and create a supportive language environment. Examples in modern media on the internet often serve as realistic learning materials, which may stimulate the interest of EFL learners and convince them of the usefulness and significance of Nursing English. Teachers can also recommend gamified learning to students, advising them to incorporate gaming elements into their learning of Nursing English. The gamification of learning has significant, positive effects on cognitive, motivational, and behavioral learning outcomes [51]. In our study, the participants confirmed the positive impact of medical games on their Nursing English learning. Through these games, students can assume the roles of nurses, doctors, or other medical professionals and tackle challenging real-world tasks. This not only enhances their medical knowledge, but also increases their motivation and enjoyment of learning Nursing English. Furthermore, teachers should provide more support to students in their process of learning Nursing English. Participants in the Sino-US diploma nursing program acknowledged several practices of American teachers that were particularly instructive. For instance, these foreign teachers provided substantial encouragement and organized various activities to stimulate students’ enthusiasm. They listened to the students patiently without interruption, even though when they were expressing themselves in broken English. The teachers assisted them in refining their responses when they encountered difficulties. These approaches contribute to creating a relaxing and comfortable atmosphere and fostering a positive teacher–student relationship. Perceived teacher caring and confirmation was positively related with students’ willingness to attend classes and academic engagement [52–54]. Moreover, a harmonious teacher-student relationship is beneficial to students’ psychological well-being and psychemotional development [55]. Students became more confident and more willing to take the initiative in confronting new challenges and tackling new problems in their learning of Nursing English.

## Conclusions and implications

This study has examined the challenges encountered by nine successful Chinese EFL nursing students in the process of learning Nursing English, along with the effective self-regulated learning strategies they adopted. It was found that these students encountered with language-related, learner-related, and context-related challenges during their learning process. They adopted diverse self-regulated learning strategies to overcome these challenges.

The rationale for our study lies not only in identifying the challenges associated with the learning process of EFL nursing students but also in uncovering the effective self-regulated learning strategies employed by successful Nursing English learners. These strategies can help future nursing students effectively self-regulate their Nursing English learning. Although the findings from this small-scale qualitative study cannot be generalized to the entire population, they do contribute to the understanding of how EFL students self-regulate their Nursing English learning.

The findings of this study have important implications for teachers, materials producers, curriculum developers, and program designers in the field of Nursing English. First, it is recommended that nursing English teachers assist students in setting specific, measurable, and attainable goals for learning Nursing English. Second, we suggest that curriculum developers and program designers include a brief overview of English word formation rules, featuring roots, prefixes, and suffixes, in the Nursing English curriculum. This inclusion will enable students to learn, reflect upon, apply, and practice these rules throughout their learning journey, which will enhance their vocabulary and terminology acquisition. Teachers should also focus more on these rules during the instruction of Nursing English vocabulary. Third, in order to cultivate a growth mindset among Nursing English students, teachers need to be mindful of the feedback they provide on student performance. When students perform poorly, teachers should avoid consoling them for their apparent lack of ability (e.g. ‘It’s OK that you are not good at Nursing English, you are good at other subjects’). Instead, they should encourage students to put in more effort and to adopt effective learning strategies. When students perform well, teachers should avoid praising their fixed ability (e.g. ‘You are so talented in learning Nursing English’). Instead, they should praise their effort and improvement (e.g. ‘Well done–I see you have put a lot of effort into your Nursing English studies’) [56]. Teachers can also share articles that highlight the brain’s ability to grow or how practicing and adopting self-regulated learning strategies improves learning, so that students can read the articles and summarize the main ideas. In addition, teachers can share stories of successful Nursing English learners, and invite them to discuss their personal efforts toward success in class. This approach may help recognize the usefulness and significance of Nursing English. It is crucial to understand that helping students to endorse growth mindsets must go beyond simply telling them to continue their efforts in learning. Rather, it is necessary to provide them with effective strategies to help them self-regulate their learning of Nursing English. Fourth, teachers can enhance students’ enjoyment of the Nursing English learning process by incorporating more imagery into their classes. For example, they could fully utilize illustrated textbooks and supplement their teaching resources with digital imagery from English TV dramas, YouTube videos, and so forth. Furthermore, providing more teacher support can also enhance students’ enjoyment of learning Nursing English.

Last but not least, we suggest that the development of additional Nursing English teaching and learning materials will contribute to the wider availability of resources in this field. Consideration of the practical application of Nursing English learned in the classroom should be integral to the development of textbooks and other teaching and learning materials. Curriculum developers and program designers in Nursing should also ensure that the curriculum and classes focus on the practical application of the language that nursing students will encounter in their future professions. Furthermore, teachers can prepare internet-generated materials for Nursing English classes or guide students to online resources, such as websites and apps. The use of authentic and up-to-date internet-generated materials can engage nursing students with topics and cognitive tasks that are relevant to their future professional roles.

Overall, we recommend that Nursing English teachers, curriculum developers, and program designers incorporate explicit strategy instruction into their regular educational practices. Such a change may effectively improve students’ linguistic competence and subject knowledge, while also enhancing their self-regulated learning [57, 58].

## Limitations and suggestions for future research

The current study is subject to several limitations. First, this study explored the challenges encountered by a small group of successful nursing students. Thus, the findings cannot be generalized across the entire population. Future research could involve a larger and more diverse range of participants, including average and underachieving students. Second, this study surveyed only students. Future research could include Nursing English teachers to provide a more comprehensive picture of this topic. Third, this study employed only qualitative methods. Future studies should consider using quantitative methods, such as questionnaires to examine the correlation between self-regulated learning strategies and Nursing English performance, or experimental designs to assess the effectiveness of explicit self-regulated learning strategy instruction in Nursing English education.

## Supporting information

S1 Data (ZIP)
