# Supplementary material for: Self-regulated learning strategies adopted by successful Chinese nursing students in the process of learning Nursing English
Source: PLoS One. 2024 Aug 8;19(8):e0308353. doi: 10.1371/journal.pone.0308353 (PMC11309511; doi:10.1371/journal.pone.0308353)
Supplement: S1 Data — (ZIP) [file pone.0308353.s001.zip › Data-English Version/Chen.docx]

**My Experience with Nursing English**

The reason why I came into contact with nursing English was when I was filling out my college application for the college entrance examination. At that time, I didn’t have much knowledge about the nursing major. Therefore, I learned a little about it from the Internet and thought that my English was good enough to be considered. I just filled it out as a backup option, and it turned out that it was a perfect match for me.

Later on, I found out that the entire teaching team was composed entirely of foreigners. I was starting to feel worried, but fortunately, we also had nursing classes in Chinese in addition to the American version.

Although I have studied the Chinese version of nursing, the class schedule was quite tight and most of the classes were American version of nursing. My knowledge of Chinese nursing was often vague and incomplete. My biggest challenge was trying to integrate the two types of nursing. As the pace and scope of instruction were different, and our time was very limited. As a result, beyond some basic concepts, much of what I learned was a jumble of two things - Chinese and English - and it was difficult to spark any connections between them. We learn both Chinese version and American version of Nursing, but could not become proficient in neither. This is a conclusion drawn by students of our major. Even though we had more classes per week than those of regular Nursing majors, it was still impossible for me to master both versions of Nursing.

In the first academic year, I felt pretty good to attend classes given by foreign teachers. However, in the second year, due to time constraints, the teachers’ pace of instruction became faster and faster (both Chinese and foreign teachers were the same). Therefore, after class, we need to put in more effort to consolidate, as exams were very frequently held by foreign teachers. Many of us felt confused about the significance of learning Nursing English. After all, we are Chinese. Why should we learn Nursing English if we are not planning to study abroad? For me, this major was also a relatively later choice, so I didn’t think too much about its significance to me. It is indeed a relatively rare major. But what I think is that since I have learned, it’s better to study hard.

Generally speaking, Nursing English is about learning nursing models of other countries, not just the nursing model in China. How to know well this ‘new friend’? For me, I believe the first step to learning Nursing English well is to have a good command of English. English listening, speaking, reading, and writing are all very important. Firstly, aside from anything else, it is important to understand what the teacher is talking about. Although there may be a lot of content in the book, nursing knowledge is not limited to memorization. Only by truly understanding it can one better digest it, especially for more abstract disciplines such as neurology and communication. A good example from the teachers’ explanation can be very helpful for learning. Secondly, just like learning Chinese, the teachers will explain some content that is not in the books during class, so you need to write it down by yourselves. There are also some points that you don’t understand in class need to be written down after being answered. In this way, English writing is also very important. Finally, although the importance of speaking and reading may not be immediately apparent, language expression can enhance communication with teachers. Only by speaking correctly and reading correctly can one understand the English spoken by teachers. Therefore, the first thing to do in learning Nursing English is to improve one’s English proficiency. I think this is the main reason why someone can’t learn Nursing English well.

Additionally, it is important to have the right mindset and firmly believe that what you are learning is beneficial. Although Nursing English is about international nursing knowledge, it is still nursing and has some similarities with nursing in China. Many people, including myself, have wondered whether it is worth learning for a domestic nursing staff who does not go abroad. From the perspective of utilitarianism, it is a good thing for learning nursing. Even though it is not popular in China, I believe that in an increasingly inclusive world culture, learning from each other is the general trend. Focusing on the nursing discipline, taking its essence and eliminating its dregs will enable better development of nursing in all aspects. And more and more people will pay attention to this discipline. In the long run, the prosperity of the nursing industry can guarantee those of us who are engaged in this industry. Therefore, even if we might feel that Nursing English is useless at the moment, Nursing English itself is still valuable.

Just like studying other disciplines, beside the right mindset, learning Nursing English demanded perseverance, especially in universities where as long as you work hard before the final exams you can pass them all. The difference is that Nursing English requires frequent consolidation. After all, it is not our mother tongue, and many contents are interrelated. If you forget them all, but work hard for several days before the final exams, no one will be able to learn it well. Daily performance scores account for a large proportion in foreign teachers' classes. In their teaching system, there will be a test at the end of each unit. This urges us to review regularly. This is actually a very effective learning mode, although we study passively. Without patience, we would likely give up halfway. Learning Nursing English was like fighting a protracted war.

Finally, it was necessary to translate and preview the professional terms throughout the textbook because there were so many of them. Many of my textbooks were annotated in Chinese. With the passage of time, it is possible to gradually reduce the amount of translation by understanding and integrating more. Before that, I still need to earnestly translate them clearly.

Because of the particularity of this major, I was fortunate enough to participate in a Nursing English competition. Sure enough, as the saying goes, ‘When you need what you’ve learned from books, you realized that you should have read more.’ It is always good to learn more. In the process of preparing for the competition, I reviewed Nursing English again and applied it for the first time in a simulated international nursing scenario. This competition is modeled after the WorldSkills Competition. But the difference is that it is more professional to take care of foreign patients with nursing skills. It is divided into 5 modules, namely assessment, communication, posture, operation, and education. Based on the competition guidebooks and the content of my regular classes, international nursing not only emphasizes accuracy and rigor in operation, but also places great emphasis on communication and humanistic care. Therefore, communication also accounts for a large module in it. And although I know the principle involved, it would be a test for me to embody it. And the teachers also put a lot of effort into correcting me. However, this is also a good thing. After all, the psychological aspect is easily overlooked in nursing. During my internship in hospitals, I saw that the common nursing situation in China is that one nurse takes care of many patients. It is difficult to achieve comprehensive care in all aspects, ignoring the psychology of individual patients and their families. This competition also reminds me that nursing is about serving the physical and mental health of the whole person, including psychological aspects. The second time I used it was during my internship in hospitals. Shanghai is an international metropolis, and although there are still some foreigners in the hospital during the pandemic. When I had to take a foreign patient’s temperature and inquire about their bowel movements, it felt like I had returned to the scene of learning Nursing English. These two things are still fresh in my memory. In my understanding, Nursing English has finally ushered in its spring.

Looking back on my experience of learning Nursing English, I didn’t learn it very well, but it is undoubtedly a precious asset. Learning is a good thing, but there is no shortcut to learning, and learning nursing English is the same. Following your own habits and pace, and with each PPT, video, exercise, and step taken steadily, you will naturally feel much more relaxed. At present, I know very little about Nursing English, but I am still grateful for the knowledge it has brought me. Not to mention the practical benefits, at least this process has allowed me to step into a different field, an attempt to explore the multiple elements of nursing. This experience is not bad.
