# Supplementary material for: Self-regulated learning strategies adopted by successful Chinese nursing students in the process of learning Nursing English
Source: PLoS One. 2024 Aug 8;19(8):e0308353. doi: 10.1371/journal.pone.0308353 (PMC11309511; doi:10.1371/journal.pone.0308353)
Supplement: S1 Data — (ZIP) [file pone.0308353.s001.zip › Data-English Version/Lin.docx]

As far as I can remember, I was exposed to English before I could even remember things, singing alphabet songs when I couldn't speak clearly. Therefore, English has never been a difficult problem for me to learn since I was a child. In addition, I started learning ‘spoken English’ from the beginning, so I didn't have any difficulties in the subsequent oral exams afterwards. It seems that I have always relied on the so-called ‘sense of language’. It wasn't until senior high school that I realized that ‘sense of language’ could not support the long articles reading question in exams, and my English scores were not as high as before. Later, I found an English teacher to make up for it. During the teaching process, he didn't tell me how to do multiple choice questions, but told me how to improve my English level and enhance my ‘sense of language. It was his teaching method that ‘It’s better to teach a man how to fish than give him fish’ has laid a solid foundation for me to learn Nursing English independently in the future.

In the first year of my university life, my English learning seems to have entered a stagnation period. Although there are compulsory courses, in order to take care of students of different English levels, the course content is relatively simple, focusing on text interpretation. However, group presentations in class are good chances for us to make us improve. Different presentation topics, from self-introduction to favorite movies, to script performances, allow us to truly use English. But this application is only temporary and limited to daily English. I especially enjoy watching American TV shows, such as Modern Family, The Vampire Diaries, and Gotham Town. I haven't missed any episodes of them. Besides class, I also have intermittent exposure to English through various means. Later on, when taking elective courses for the convenience of earning credits, I chose Nursing English, which I had confidence in, and only then did I officially come into contact with a different professional English. In my classroom learning, I found that the Nursing English words were very complicated. For me, reciting a new Nursing English word was like remembering a lot of unintelligible codes. While learning Nursing English, I gradually learned the word formation rules. I used prefixes and suffixes to distinguish Nursing English words, such as anti- means confrontation, cancellation, inhibition and dissolution, veno- means vein and vaso- means blood vessels, etc. But with too many words and too many classifications, most of the time I still choose to seek help from translation apps. In fact, Nursing English is often not so flashy and inaccessible. It is more about communicating with patients, and it is not much different from the daily communication learned before, only the application scenarios are different. Later on, by chance, I learned about the Medical English Proficiency Test (METS) through banners in campus. It was mostly a test-oriented learning, where I familiarized myself with the exam routine by practicing the designated textbooks and eventually passed the exam.

Nursing English truly filled my learning life since the winter vacation of 2017. At that time, I passed the first school selection for the Health and Social Care Program of WorldSkills competition. The first learning task of the winter vacation was to read a large number of Nursing articles in English. As someone who is accustomed to using translation software, I spent a lot of time on this task. The continuous learning throughout the winter vacation gradually helped me regain my ‘sense of language’. After the start of the next school year, in addition to professional operational skills, Nursing English is also a key focus in training for the WorldSkills Competition. The problems of ‘not being authentic enough’, ‘being cold and aloof’ and ‘cannot express oneself properly’ are all completely exposed in front of my English teacher. Moreover, I frequently spoke Chinglish. These are corrected one by one, such as my pet phrase ‘well’; my insincere saying ‘Everything will be fine’, or the ambiguous phrase ‘Am I clear?’ and long attributive clauses. In order to change these habits, we practiced over and over again. In the training room, we were like actors who constantly failed. We repeated again and again until we optimized and stumbled through the selection process for the selection competition of Shanghai.

Nursing English is useless in general clinical work. I deeply felt this during my internship in a hospital during the training period. There were very few textbooks and learning resources related to Nursing English available in China. Most of the materials we refer to were all in English, which were thick and heavy, and could not really arouse our interest in learning. However, it is still of great help in improving nursing skills in daily study. In the competition, in order to benchmark foreign nursing skill standards, we would learn from online video resources, most of which come from YouTube and involve some professional vocabulary. Without learning Nursing English, we may not be able to understand it. In addition, in the WorldSkills Competition, we needed to explain medical terms to patients in simple English, necessitating English-to-English translation. Expressing these professional terms briefly in English was also a challenge. Before the competition, I would first have a comprehensive understanding of the disease, and then tried to explain it in a vivid way, such as DVT (deep vein thrombosis). I would use my hand to compare a circle to a blood vessel to explain it, so that patients could listen and see at the same time to help them understand. The foundation of English-to-English translation requires a thorough understanding of words. Therefore, during the training process, the teachers require us to give an English presentation on the relevant diseases and be able to answer their questions. Although it may be tedious, it is effective. Next is the issue of language habits. In the competition, we need to assess the patient’s condition, guide their rehabilitation exercise, comfort them, and solve some social problems. Due to time constraints, we need to express ourselves clearly in short sentences. Perhaps because we learned too much grammar in high school, I always like to use long relative clauses, and sometimes even get caught up in them. Later on, I learned to use some short sentences, such as look straight, hold tight, left right, etc. when patients are doing rehabilitation exercises. They can quickly understand and act. At the same time, it is easier to remember. But not all problems are so simple and crude. When patients express negative emotions to us, we need to give them the correct response and show them our sympathy. At the beginning, I will use ineffective comforting sentences such as ‘Don’t cry’ and ‘Don’t be sad’, but in the course of teachers’ guidance and study afterwards, we will use I understand and I’m sorry to hear that instead. I will also use open-ended questions to some patients who need to confide in me to tell me their needs.

In this way, there seems to be no problem that needs to be solved. My Nursing English should be sufficient. But in fact, the most outstanding problem is the pronunciation and intonation. At the end of a 30-minute case, the teachers were usually sleepy and couldn't withstand my hypnotic tone. This is really difficult to change. Being too nervous and lacking authenticity may be the reason for the problem. After clinical learning, there was some improvement. In the subsequent training, we went to United Family Healthcare, where we were exposed to the whole English working environment, including work shift, communication with doctors and patient. In the process of clinical learning, I found that the communication among the teachers was very natural and smooth, while also paying attention to cultural differences, such as the tolerance of pain. Compared to the clinical practice of Nursing English at United Family Healthcare, the Nursing English I learned from textbooks was not very practical.

After ‘retired’ from the WorldSkills competition, I seldom use Nursing English. More likely, I just spit it out for fun in American medical dramas. But the gains from studying Nursing English can still enrich most of my university life. In this process, my understanding of Nursing English is not only complex and sophisticated professional vocabulary, but also the sentences used in daily communication with patients and every sentence we say in the nursing process are within the scope of Nursing English learning. Nursing English should be warm and emotional, allowing us to understand patients while also healing them.
