# Supplementary material for: Self-regulated learning strategies adopted by successful Chinese nursing students in the process of learning Nursing English
Source: PLoS One. 2024 Aug 8;19(8):e0308353. doi: 10.1371/journal.pone.0308353 (PMC11309511; doi:10.1371/journal.pone.0308353)
Supplement: S1 Data — (ZIP) [file pone.0308353.s001.zip › Data-English Version/Liu.docx]

**My Review of Nursing English Learning**

I am honored to be invited by Mr. Wang to review my English learning over the past decade. I came into contact with the discipline of nursing for a long time. When I was 15 or 16 years old, I chose nursing as my major and studied at Shanghai Health School. At that time, I studied nursing in Japanese. Apart from the first academic year's basic course of English, the following two years were mainly Japanese courses. At that time, I was worried that if I didn’t learn Japanese well, I might have taken my English studies astray. The popularity of Japanese English is evident to all. Although I chose nursing as my major with a focus on Japanese at the time, I never gave up on English learning. In the library, I actively search for English grammar books and comics with English conversations. I am also interested in English documentaries. However, the beginning of change still lies in the opportunity of independent enrollment exams for the college entrance examination. At that time, I needed to take the exams of three basic courses-Chinese, mathematics, and English. I hadn't systematically studied English for two years. Fortunately, my passion for English has not diminished. My love for the language has always been there. This exam has also motivated me to continue learning English well. Thanks to a short period of extracurricular classes and exam paper practice, I also achieved a top 15% and A-level rating in the academic proficiency exam. This low value ‘exam’ inspired me at that time and made me feel like I needed to pick up English again.

The real learning of Nursing English began in my freshman year of college, when the textbook was New College English Course. However, there were also a series of elective courses in college. In my freshman year, I took the English Salon course, which was taught by foreign teachers of our school. There were 100 students attending this class. My friends and I always sat in the front row. Foreign teachers will focus on topics such as dietary habits, culture, art, movies, and festivals, greatly enriching my understanding of English. In my sophomore year, I chose Nursing English as an elective course, which also improved my professional English through common medical vocabulary, diseases, and conversations in daily hospital settings. I participated in the 2019 National College English Competition with a trial-and-error mentality and won the second prize. In the same year, I took the Medical English Test (METS) Level 3. I have always believed that nursing is a helpful discipline. If you want to progress in nursing, you must keep updated with both domestic and international nursing trends. At the same time, there are many drugs and treatments that may have just started in China, but have become more mature abroad. This requires you a good command of Nursing English. We learned vocabulary and grammar through rote learning. However, we must still adhere to certain scientific principles. For instance, prefixes, suffixes, and roots often represent the most fundamental meanings of words. For example, prefixes such as anti-, homo- and gastr-, suffixes such as -logy, roots such as ‘enter’ can be combined to form gastroenterology, which is translated as 肠胃学. I borrowed a book named Medical English Terminology (the second edition), compiled by Professor Mingshan Yang from Shanghai University of Traditional Chinese Medicine Press. This book introduces the relevant history of medical terminology, five basic characteristics, key word formation rules, special pronunciation rules, chronology, words frequency, and the most commonly used word formation tables. It is a very useful medical English textbook. There are many compound words in Nursing English or medical English. These parts need to be pieced together like parts. At the beginning of learning Nursing English, I found it extremely difficult. Medical terms are usually unfamiliar, exceedingly long and challenging to pronounce. While I recall their meanings when reviewing them in the textbook, they seem like entirely new words once the book was closed. This experience was common among my classmates. I used flashcards to aid my memorization of new words. Usually, A4 paper cut to 1/16 is used, with medical vocabulary written on the front and the corresponding meanings written on the back. I personally think this method is still very good for memorization, as it has helped me remember many difficult words.

In Nursing English learning, some internal and external factors, such as the lack of motivation and authoritative learning resources, have impacted our effective learning. This type of problem is encountered by both young students and seniors who have already entered the medical profession. The methods of learning Nursing English and learning general English are very similar, and it is a special and general relationship. By reflecting on my own experience, I think the most important aspect of learning Nursing English is still the memorization and application of professional English vocabulary. Besides, speaking and writing are also very important. In China, the medical environment where English was spoken consisted mainly of foreign-funded hospitals and VIP clinics of hospitals. However, with the advancement of information technology, it is now possible to learn the most advanced Nursing English courses without leaving home through online learning and online MOOCs. The key was to take the first step learning Nursing English and stick with it.

Learning is for applying what you have learned. In order to enhance my practical skills and professional competence in Nursing English, I also participated in the Shanghai Selection Competition of the Health and Social Care program of the 46th WorldSkills Competition during my university years. This competition is known as the Olympics of the skills, testing the contestants’ English communication skills, professional nursing skills, and problem-solving abilities. Through 5 rounds school selection, I was selected from hundreds of contestants. Under the guidance of my nursing teacher and my Nursing English teacher, I competed in the competition on behalf of my school and won second prize in the Shanghai Selection Contest of the ‘Health and Social Care Program’ of the 46th WorldSkills Competition. This is also my first time participating in an all-English nursing competition, which has opened a new chapter in my English nursing career. After participating in this competition, I joined the Shanghai training team of the WorldSkills Competition. Through studying English corpuses and interning at international hospitals, my practical experience in Nursing English has been further improved. Two years later, I teamed up with my junior schoolmate from the Sino–US nursing program. We participated in the 2021 Nursing Skills Competition for Domestic Medical Colleges, which is also a nursing competition for English speaking patients. I won the first place in the school group and also received the individual operation award.

In preparing for the competition, I could not have succeeded without the help of Nursing English teachers. Their support also strengthened my motivation and confidence to continue learning Nursing English. I will continue to work hard and believe that every step counts.

In summary, my personal goals for learning Nursing English are to read English nursing literature, communicate freely in daily medical scenarios with medical teams, and fully express my opinions on foreign medical related forums without English becoming as obstacles. At present, my vocabulary and Nursing English proficiency are still in the development stage, and there is still some distance to my goals. I did believe that I would gradually make breakthroughs in the future through further study and the accumulation of knowledge and experience. The learning of Nursing English is long and full of challenges and opportunities. May we all firmly use scientific English learning methods to lay a solid foundation, break through limitations, and make further progresses in Nursing English learning.
