# Supplementary material for: Self-regulated learning strategies adopted by successful Chinese nursing students in the process of learning Nursing English
Source: PLoS One. 2024 Aug 8;19(8):e0308353. doi: 10.1371/journal.pone.0308353 (PMC11309511; doi:10.1371/journal.pone.0308353)
Supplement: S1 Data — (ZIP) [file pone.0308353.s001.zip › Data-English Version/Shan.docx]

I think that I am an ignorant person who has survived until now based on the knowledge gained from the nine-year compulsory education. After being admitted to the Shanghai Health School, my classmates around me were more or less capable, including those from the prestigious Shanghai High School Affiliated Middle School, academic overachievers who obtained many certificates, and those who were proficient in music, chess, calligraphy, and painting. I was somewhat inexperienced. Therefore, I had to work hard. The curriculum of Shanghai Health school can be roughly divided into two categories: professional courses and basic courses. My major is nursing in which professional courses account for 2/3 of my study, and the remaining 1/3 is basic courses such as Chinese, mathematics, and English. The basic courses are is not difficult, but there are still students who score less than 10 for English courses. Therefore, basic courses are divided into different levels. And I was placed in Class A. There is a classmate who has influenced me a lot, let’s call her ‘top student’. She comes from a prestigious affiliated high school, and her English is considered elite. I naturally admire and yearn for the strong. A few months after the start of the school year, the school opened a Nursing English class, and the ‘top student’ signed up. How could I fall behind? Mr. Wang appeared on my rather bumpy learning journey at this moment. As a beginner in Nursing English, the class is full of enthusiasm for learning, as if one cannot leave this classroom without mastering it. Nursing English is pretty easy. Nose, eye, and ear, Nursing English is so simple. It’s not difficult to ask the patient’s name, and all we transcribe is various numbers related with patients. After the first class, a few students sneaked out the back door. In the second class, there was a sudden change and it is about the study of word roots. Mr. Wang spoke eloquently about ‘acid is 酸’, while the top student listened attentively, but I started to feel confused and didn’t understand. At that time, I was studying Level 1 Nursing English, which had 200 main vocabulary words. When I didn't understand, rote memorization became very useful. After all, I had used this method since I was a child. I set a small goal – to recite ten words each day. This lasted for six days. When I looked back at the first word I memorized, I felt both familiar and unfamiliar, I unexpectedly failed the classroom test. I freaked out because I failed the quiz. I was certain that I had carefully studied the new words. Why was my score still so low? The enthusiasm for learning has been extinguished quite a bit. After several classes, our Nursing English class had dwindled to one teacher and ten students. The top student was still proficient in digesting the knowledge of each class, while I still listened to Mr. Wang’s lectures with only a partial understanding. The more difficult the class, the fewer people there are. By the time we got to the word root ‘hyper’, only four or five students remained. My classmates’ departure made me waver on the verge of skipping class, and I was ready to give up.

The school has started the selection process for the annual International Nursing Skills Competition, and I have also participated in the selection process, following in the footsteps of the top student. During the interview, I felt the varying levels of the contestants. The senior students spoke confidently. While reflecting on myself, I blushed and stuttered before I could say anything. After preparing for a long time, I could only speak out the simplest words, ‘I am, I am...’. Of course, I was eliminated. Afterwards, I also kept an eye on the training trends and watched the matches. I admired the calmness of the strong so much, and I reflected on my learning progress. One reason for my limited understanding is my insufficient vocabulary. My vocabulary was tested as only around 2000, which may be sufficient for daily communication, but it is far from enough to deal with nursing knowledge. The second reason is that I used the wrong method. The progress of modern technology brings us different resources. On the internet, I can see the experiences of countless predecessors, such as the Tomato Learning Method, the Ebinski Forgetting Curve. I can also read books in English... Every time I see a new method, I can’t wait to practice it on myself. Then I realized I had learned too much without proper assimilation. Moreover, my goal was too ambitious. I must find my own learning method. As the saying goes, interest is the best teacher. What things can make me feel happy? It’s a movie with ups and downs, a game full of passion, and an unknown new thing. I would shadow read my favorite passages aloud when I watched American TV series. Because of my major, I watched more TV shows related to healthcare, such as ‘Grey’s Anatomy’ and ‘House M.D.’ The first word I remembered was cirrhosis. At the same time, teachers on YouTube would illustrate and explain the various organizational structures of the human body. You learned the new vocabulary when you enjoyed these videos. The medical puzzle game developed by the University of Hong Kong made us memorize medical terms subconsciously while doing brain teasers. English for special purposes is just a branch of English. It is essential to increase vocabulary. I have experimented with numerous learning apps, such as ‘LAIX’, ‘Shanbay’, ‘Duolingo’... Baicizhan is the most effective one I have used. The moment I turned on my phone, the words with pictures popped up and I use fragmented time to memorize them. I actively participated in various English competitions both on and off campus, from failing to winning the top three prized. I insisted on attending Nursing English classes from Level 1 to Level 4. There were only four or five students persisted to the last class. Time will prove everything. Within two years, my vocabulary expanded to 7,000 words, and I advanced from a beginner to an advanced Nursing English learner.

At the beginning of the new semester, I moved to a new campus. I participated in the English corner at the Pudong campus and met David, the teacher who led me to experience the charm of English. Every Monday afternoon, we all felt the charm of English together. The accents from all over the country spoke about our love for English. There was a student from Henan Province who left a vivid impression on me. His spoken English was not fluent, but he was stumbling, not afraid of the stage at all. As soon as he spoke, it feels like he is performing a stand-up comedy, which made the whole class burst into laughter. He would gesture the words he didn't know. Everyone could understand him. His confidence drove everyone’s interest in learning. Being in the same class with such a classmate is really a blessing. And I am more willing to learn! English is an art that allows people from all over the world to recognize and fall in love with it. Here, I have also undergone a transformation in my mindset about learning English. English is not just words composed by 26 alphabets, neither it is grammar related to subject, verb and object. It is the communication between people and the collision of cultures that makes me braver and more confident to express my thoughts in English. At the same time, my nursing internships in various hospitals and interactions with patients have taught me the importance of communication. A sentence can have thousands of meanings. At the beginning, I started learning Nursing English to emulate the proficient learners. Gradually, I became the proficient learner among my peers. Learning has allowed me to constantly analyze myself. I could feel the enjoyment of learning Nursing English. From blindly following to self-improvement, learning will always give you answers.

The selection competition for the WorldSkills Competition was held when spring came. Over the last three years, my efforts have been witnessed by teachers and my professional performance has also demonstrated my strength. After successfully being selected, communicating in a pure English environment has greatly improved my speaking skills. In the past, when I spoke English, I would always have subject verb reversed. I spoke too fast, and often paused due to not knowing the English word. I was at a loss, my mindset needed to change first. I needed to learn how to comfort myself. After all, I am not a native English speaker. There will always be unfamiliar words, but I should not be nervous. I can find words with similar meanings to make simple expressions. Secondly, I should not always try to replace them with advanced vocabulary. Easy to understand is the best way to express myself. I always speak too fast, Until I think from my perspective, I just realized that speaking fast doesn't necessarily represent my English ability, making others understand is the key. Facing patients, while demonstrating the professional skills of a nurse, care and understanding are particularly important, making patients to feel the warmth of language. The comprehensive training has also solidified my nursing foundation, The efforts of my two preparation partners have further stimulated my enthusiasm for learning. It is not limited to nursing courses in China. My tutors have sought nursing courses in other countries for us. The collision of Eastern and Western ideas has made me feel the unique charm of nursing, and at the same time, I also feel the desire to acquire different knowledge and learn foreign knowledge.

After the competition, I joined a study tour program at a Finnish university and decided to go abroad to experience nursing firsthand. Compared to nursing in China, Nordic nursing is very mature and more developed. The Nursing English textbooks were too blunt. But this tour to Finland deepened my understanding of Nursing English. The 8-month nursing internship also allowed me to combine English with work in my daily life. They were a father and son from Brazil. The son had obvious injuries to his left foot and was struggling to walk. They were anxiously waiting at the entrance of the emergency orthopedics department. The emergency doctor was dealing with another fractured patient. The pain caused the young man to be unable to hold on and almost fell to the ground. His father anxiously shouted. This scene was not uncommon in the crowded emergency room. With different languages and unbearable pain, the father and son sweat profusely, As I saw this when I was about to finish my work, I borrowed a wheelchair in the lobby and explained the situation to the doctor. While he was treating the fractured patient, I first asked the young man about his chief complaint. This was also the first time I had used Nursing English in my daily life, such as what is your name, where are you from, where do you feel uncomfortable, how much pain is there, how long has it lasted, calm down, take some deep breath. After the doctor finished his treatment with the fractured patient, I informed him of the information obtained, which not only reduced the doctor’s repeated evaluation but also shortened the time for the patient to receive treatment. This experience greatly strengthened my belief in continuing to further study Nursing English.

After graduation, I worked in the ace department of a 3A hospital. Here, I had more opportunities to apply Nursing English into practice, I designed health education in English, served foreign patients, introduced disease-related knowledge, completed a series of processes from admission, treatment to discharge, and taught internship students. Learning never ends. In order to complete the academic tasks of the department, reading English literature has become a daily requirement. The obscure and lengthy Nursing English vocabulary was extremely difficult and challenging for me. It was only then that I realized my lack of professional knowledge and my learning was far from enough. I also enjoyed it a lot. Reading English literature has briefly improved my English in a certain stage. Usually, after reading a piece of literature, the blank space was filled with Chinese translations. Due to the professionalism of medical vocabulary, I encountered many difficulties in translation. Google’s machine translation made the language completely incomprehensible, Therefore, I had to search for dictionaries word by word for translation and then polish them together. The process was very difficult, but the result was good. When I kept reading papers on the same disease, I became particularly sensitive to the vocabulary of some diseases. Repeated memorization also increased my vocabulary, reduced reading time, and increased the amount of reading. Major hospitals will organize summits. I cherished every precious opportunity. During the summit, experts from all walks of life will present their research results. I cannot help admire them so much. With the desired goals, there is more motivation to continue moving forward.

Ordinary people tell ordinary stories. If I had chosen to give up learning Nursing English with others at that time, I would not have had so many memorable experiences. My English learning journey was also quite bumpy, with numerous failures. But hard work and time will tell me the answer. For me, having a clear goal motivated me more. What’s more, interest make me more passionate about learning, tireless and immersed in it. I am honored to have met teachers who are good at teaching and led me to explore the charm of language instead of exam-oriented education. Encountering excellent classmates not only changed myself, but also influenced me. After encountering a settled self, I started to learn for myself and strive for myself.
