# Supplementary material for: Self-regulated learning strategies adopted by successful Chinese nursing students in the process of learning Nursing English
Source: PLoS One. 2024 Aug 8;19(8):e0308353. doi: 10.1371/journal.pone.0308353 (PMC11309511; doi:10.1371/journal.pone.0308353)
Supplement: S1 Data — (ZIP) [file pone.0308353.s001.zip › Data-English Version/Tang.docx]

When I first entered university, I first came into contact with Sino-US nursing courses, and thus I realized the importance of Nursing English. The Sino-US nursing program not only requires a solid grasp of nursing knowledge, but also proficiency in English oral communication and mastery of certain American practical and comprehensive skills in future nursing work. Throughout my university years, I have consistently pursued autonomous learning of Nursing English. Mastering Nursing English has provided me with clear goals and directions for the future, and has also played a guiding role in my career development.

The classes given by foreign teachers in universities are significantly different form the classes in my high school. Firstly, the content of instruction is expanded with a small amount of vocabulary and images on the slides, replacing the traditional teaching mode in high school. We need to supplement the key vocabulary we hear in the notes distributed in class. This is a test of our focus and comprehension ability. For me, sometimes in class, I may feel confused. For example, when I haven’t fully understood the previous part, the teacher immediately shifts to the next part. Secondly, in classes in universities, emphasis is placed on listening and speaking. Foreign teachers do not allow us to discuss in Chinese during group discussions, but communicate the content leant in English. The foreign teachers encouraged us to speak English. In order to fully mobilize our enthusiasm, foreign teachers organized activities such as classroom debates, watching videos, and group quizzes. The topics that can be discussed in class are also diverse and selective, covering movies and TV dramas, Chinese cuisine, Chinese history, Sino US differences, etc. These topics are very close to the lives and hobbies of young people, allowing us to speak freely and have something to say. What touched me was that they would listen patiently without interrupting us, regardless of our English level. They would help us refine our answers when we encountered difficulties. When I heard their comment ‘good job’ again and again, my confidence grew, and I became more willing to take the initiative independently to face new challenges, and I could even give a public speech on the platform later.

I have laid a solid foundation in English and mastered English grammar in high school. However, the absence of a specific English communication environment led me to develop the bad habit of ‘dumb English’. Opening my mouth and immediately converting Chinese in my mind into English has become a major obstacle to my English learning. Therefore, it is still difficult for me to adapt to the all-English classroom in college all at once. Fortunately, when I encountered problems in my study, I could ask the foreign teachers for help anytime. They warmly invited me to join them for dinner, providing more opportunities to practice my speaking English. As my friendship with the teachers grew, I naturally became less shy about speaking English.

During the first few weeks of school, I decided to catch up and immediately set learning goals to keep up with the course. After returning to the dormitory every day after class, I persisted in autonomous learning. In addition to completing the day’s homework, I also followed my study plan. I would preview the content that would be taught the next day, comprehending the main idea, and grasping the theme. I looked up unfamiliar medical terms and new words in advance and annotated them to strengthen my understanding, which also deepened my impression of the words. After studying a chapter, I made a learning outline, reviewed the key points of each chapter, and used a mind map to summarize what I had learned. When studying medical anatomy and medical terminology, I recommended that my classmates use flashcards to improve recitation. Initially, new words were sorted by roots and affixes, and related words were written on the same card. Before breakfast, I reviewed the vocabulary using the flashcards that I had brought along. These are all good ways to make up for one’s shortcomings with diligence, and the results are also very significant. In summary, while college English classes provide us with considerable autonomy, they also bring great challenges.

Later, I was fortunate enough to participate in the Chronic Disease Care and Health Promotion project of the 2018 Shanghai International Nursing Skills Competition, during which Nursing English played a great role. This competition is based on simulated cases and tasks, with foreign teachers playing the role of standardized patients. Good nurse patient communication and standardized nursing operations are both important aspects of nursing in China and the United States, which is a rare opportunity to transform what has been learned into practice. During the formal training, nursing teachers and English teachers led our team members to study the details of each operation, carefully designing each movement, thus making the operation more correct, standardized, compact, and aesthetically pleasing, as close to clinical practice as possible. What remains unchanged in both Chinese and American nursing operations is humanistic care, and the concept of CICARE in American nursing has always been present throughout the entire process, which was imprinted in my mind. It requires us to introduce ourselves before any nursing operation, explain to the patient and obtain their cooperation, and then check. We attach great importance to the patient's privacy and care about their feelings. At the beginning of the drill, I was too focused on writing a good manuscript, ensuring its completeness and correctness. Later, my teachers discovered this problem. They corrected my thinking and provided me with clear direction. The teachers pointed out that asking patients questions is a two-way communication process, and we should have a positive listening attitude, rather than just focusing on expressing what we want to say and ignoring the patient's feelings. Next, my partner and I immersed ourselves in repeated practice, from initial process training to strict control of time, and then to drills to deal with various unexpected situations. Finally, we were so lucky that that my partner and I won the first place in the competition.

As learned in nursing communication class, timely feedback is necessary after receiving a patient's response, which can be achieved through language, eye contact, facial expressions, body movements, or repeating what the patient says. The concept of superior, which was mentioned in the critical thinking class, was greatly applied during the competition. Nursing assessments and measures were implemented in an orderly manner based on patients’ condition, which benefited me greatly in future nursing work. This experience has also greatly improved my English proficiency and various abilities.

With the increasing number of foreign patients, clinical nurses need to have excellent English communication skills to quickly and accurately collect medical history, grasp the condition, and bring convenience to medical work. Nursing English follows closely in work, and when encountering difficult problems, one can also consult literature, broaden their horizons, and solve problems. Therefore, I hope to hone myself through learning Nursing English, so that I can better align with international nursing concepts, do my job well, and develop towards my area of expertise. I firmly believe that success can only be achieved through gradual and down-to-earth steps, as one must feel inferior when climbing high and approaching distant places.
