# Supplementary material for: Self-regulated learning strategies adopted by successful Chinese nursing students in the process of learning Nursing English
Source: PLoS One. 2024 Aug 8;19(8):e0308353. doi: 10.1371/journal.pone.0308353 (PMC11309511; doi:10.1371/journal.pone.0308353)
Supplement: S1 Data — (ZIP) [file pone.0308353.s001.zip › Data-English Version/Wang.docx]

**My Experience of Sino-US Nursing Program**

When I was in junior high school, the college entrance examination system was still 3+1, and I chose physics. My English has always been poor, and I have never passed any English exams in senior high school (in fact, the closest score was 57). During the college entrance examination, I was admitted to Sino-US nursing program taught entirely in English by chance. At that time, I felt a lot of pressure. If I couldn't understand the content of American teachers’ classes, what was the point of me staying here? But due to my strong mind, I did not give up and instead firmly believed in the education given to me by Shanghai University of Medicine and Health Sciences. I spent all my time on English learning, either in spoken English or professional English. Repeating it over and over again every day, from having to gesture when speak to American teacher, to gradually being able to express myself more fluently. As someone with poor English foundation but who wants to improve, the hard work is self-evident. Here is a brief record.

First of all, the most important thing is our mindset. We should always believe that as long as we work hard, we can succeed. You can hold a humble heart that although your current position is low and relatively poor. There will definitely be a turning point if we persistently work hard and gradually. Although my foundation is not good, I always believe that I can do it well and not inferior to anyone. Sometimes I even have a bit of blind confidence.

Indeed, there are numerous difficulties in learning Nursing English, including technical challenges such as the complexity and difficulty in memorizing medical terminology. Or because all professional courses are taught in English, it is inevitable to involve professional vocabulary. In fact, I believe that to learn a subject through English may be a shortcut to improve my English. Because you also do critical thinking when you learn, and this often achieves twice the result with half the effort. In addition, the curriculum of the Sino-US nursing program at Shanghai University of Medicine and Health Sciences is very reasonable. In the early stages, basic terms such as Medical Term were taught, gradually integrated into the teaching of professional courses. The basic vocabulary learned in the early stages often appears in later professional courses, making your learning effect better. At the same time, the American teachers made me feel what standard English is like throughout the course (my teachers’ pronunciation was still accented in high school, which often made me uninterested). Although American teachers also focused on teaching grammar, they did not overly emphasize it. This approach helped to create a relaxing atmosphere, enhancing our enjoyment of learning. Moreover, some classroom practices of American teachers, such as not requiring students to stand up to answer questions and giving small gifts as incentives, narrowed the distance between students and teachers. They always remembered our birthdays which also promoted a good teacher–student relationship and facilitated the teaching and learning of Nursing English.

Of course, not every student in college is passionate about learning. Some not only do not learn on their own but also do not allow you to learn, or they learn on their own but do not allow you to learn. They seem to have a natural aversion to learning, an innate contempt for those who study. They will influence you with various ways in life. for example, they may say that you are so awesome, why didn’t you get admitted by any undergraduate programs in the college entrance examination. Why are you pretending like that? You’re just pretending to be a top student, imagining that you’re a real top student. If you do slightly worse on an exam, they will say, ‘Didn’t you work very hard? Why didn’t you do well?’. There is also behavioral influence. For example, intentionally distancing you from others, only socializing with friends who play games with themselves, or playing games until early in the morning, intentionally shouting loudly with the microphone on, not going to class early in the morning, yet you still have to drag your tired body to class. When facing these situations, we need to adopt some strategies that not only solve the problem, but also avoid conflicts. Due to conflicts, not only will it waste the counselor’s and our own time, but those so-called classmates will also think that you are complaining and escalate the situation. Therefore, I conducted an investigation, asking about the rest time in each dormitory. After obtaining some information, I screened and asked if there were any available beds and if they were willing to have me come over and rest at night. Later, my classmate in other dormitory allowed me to rest at night and we all go to bed very early, and the problem was solved. Actually, what I want to express is that people must learn to turn negative factors into positive factors. For example, when someone makes you unhappy, you can turn negative emotions into motivation to work hard. In fact, this is also the case. After I was admitted by undergraduate program, my roommates became much more considerate. When you rest, they put on headphones and keep the environment quiet. Learning English is often influenced by multiple factors, but it is also crucial to be able to effectively address external negative factors.

The most important learning activity for me was reviewing the Nursing English content of the day bit by bit after class every day. I felt extremely fulfilled when reviewing the nursing textbooks in English. I secretly turned on the air conditioners in the classroom. I reviewed the teachers’ slides several times and repeatedly wrote down the content until I could roughly summarize the lesson for the day. I believe that we all need some lonely time, which makes us grow. I can clearly feel that as I deepen my learning, my reaction to medical terminology becomes faster and my memory is also increasing. And for me, participating in competitions or showcasing in some activities afterwards is a process of output, far less helpful than learning English alone at that time.

In addition, I have a very good relationship with foreign teachers such as Paul and Dave. It has been four years since I graduated, and we still often eat and chat together. When we were in school, we often had lunch together, talked about our families, and our future dreams. Don’t be shy to speak up and feel that your speaking skills are not up to par. In fact, most Chinese around you cannot identify any errors in your spoken language. As long as your pronunciation is slightly standard, they will think you are amazing. Therefore, there’s no need to worry too much. As long as we can communicate and express ourselves, we can be more confident. After all, we can also memorize some ancient poems. If we speak these up, the foreign teachers will also be shocked. There is no need to overly care. We should create an English environment to put ourselves in an excellent English learning environment. We should learn the language, but not just learning the language. We should also make friends with foreign teachers, engage in casual conversation, integrate language learning into daily life, watch American TV dramas, listen to English songs, and imagine yourself as a foreigner. To understand the surrounding environment, it is important to be intuitive. For example, instead of translating the word ‘book’ into Chinese, you should immediately conjure up an image of a book. Your progress in learning Nursing English terms will be fast if your imagination is engaged.

My nursing English learning has enabled me to communicate effectively and my vocabulary is basically sufficient for daily needs. Although I have also taken some exams such as the College English Test Band 6 and the National Medical English Proficiency Test Band 4, there is still a gap between me and the English majors. This is particularly evident when I am writing English papers (which require a relatively complete logic and unique expression). But there are always solutions to everything. I downloaded some highly influential SCI papers to imitate their expression and sentence patterns. I have gained some rewards after a long time. I have achieved my English learning goals during my college years, but currently I still have a lot to learn in writing English papers. The learning of language is a long-term and continuous process, and I am still in the stage of learning at present. My learning methods are effective to some extent. But my English can only meet daily communication needs. There are still many shortcomings in scientific research exploration and paper writing. I am still exploring the methods involved. I hope to improve my learning strategies through continuous thinking and strive to get closer to my goals as soon as possible.
