# Supplementary material for: Self-regulated learning strategies adopted by successful Chinese nursing students in the process of learning Nursing English
Source: PLoS One. 2024 Aug 8;19(8):e0308353. doi: 10.1371/journal.pone.0308353 (PMC11309511; doi:10.1371/journal.pone.0308353)
Supplement: S1 Data — (ZIP) [file pone.0308353.s001.zip › Data-English Version/Wu.docx]

**Promote Learning through Competition and Learn for Practice**

I started to learn English from the fifth grade of elementary school. I was very interested in this foreign language at first. I liked the 26 alphabets, the writing and pronunciation and its simplicity. I also enjoyed listening to English songs, and watching American TV shows. As the saying goes, interest is the best teacher. I gradually developed a good sense of language and standard pronunciation through in class education and daily life, which laid a solid foundation for my later learning of Nursing English.

I am an undergraduate of nursing program. I continued to study general English in university I was introduced to Nursing English through competitions in the second semester of my sophomore year. I registered to participate in the campus selection competition for the ‘WorldSkills Competition - Health and Social Care Program’. As the WorldSkills Competition is an international event, the language of the competition is English. And this is my first time participating in an English based competition. I was shocked when the written test paper for the first round of selection [for the WorldSkills Competition] was placed in front of me. It was filled with multiple-choice questions in English and a multitude of professional medical terms. The pressure was immense. I completed the exam, and luckily, I passed. In order to cope with the upcoming practical oral communication, I began to learn and accumulate medical vocabulary, and memorize relevant sentence patterns. Despite all my preliminary preparations, I still felt overwhelmed, as if you had accumulated a lot but couldn’t output or didn’t know how to output. My initial thought about Nursing English was that it was nothing more than professional medical terminology combined with daily English communication. For daily communication with patients, we just need to translate the conversation into English. This understanding makes my conversations with patients appear awkward, with frequently used medical terminology. The communication with standardized patients is not as smooth as expected. Later on, Later, I began consulting with foreign teachers and English teachers at our school, and learned the AIDET model of nurse patient communication, which gradually sparked my interest in Nursing English and helped me understand that building a good nurse patient relationship requires a language template. I have also gradually gained an understanding of therapeutic communication in English, which expressions are more appropriate or ambiguous. Moreover, I have also learned some more authentic English expressions.

At first, based on my previous English learning, when faced with patients' complaints, I would immediately answer, ‘Don’t worry, everything will be OK!’ This expression is very common, but perhaps more applicable to friends in daily life. In Nursing English, I realize that this type of expression may not be the best answer. Patients will not be relieved by your comfort ‘don’t worry’. Instead of providing ineffective comfort, it is better to sincerely describe what you see and feel, and then encourage patients to express their inner concerns. And now, I can say, ‘Well, you look pretty low, what made you feel this way?’, ‘Those feelings you have are totally normal.’ Through the competition, I gradually understood that Nursing English is not just nursing plus English, but rather using English as a medium to integrate and convey the professionalism and humanistic care of nursing.

In learning Nursing English, the intimidating terminology, enormous vocabulary, and the overall difficulty in listening, speaking, reading, and writing made me anxious, especially when trying to memorize terminology. Fortunately, when I began to worry about memorizing these professional words, I came into contact with the compulsory course Nursing English. I realized that medical vocabulary follows specific word formation rules when foreign teachers explained the roots, prefixes, and suffixes. By dividing and reformatting each part, I could learn even the most complex and lengthy medical terminology. As the saying goes, a good memory is not as good as a bad practicing. I repeatedly dictated difficult words each day to deepen my impression. I used reference materials like the Illustrated Guide to Medical Terminology to assist word memorization with the help of pictures. With the word formation study of medical vocabulary, it is easier for me to learn nursing textbooks and analyze cases.

After the problem of vocabulary learning is solved, the next problem comes is English-to-English translation, which is how to explain a term to patients in simple and understandable sentences. I remember during my first practical exercise, one task was to help patients prevent ‘DVT’, which stands for deep vein thrombosis. When I started this task, the patient asked, ‘What does this mean?’ and my explanation at the time was ‘DVT means deep vein thrombosis’. But this did not solve the patient’s confusion. This incident made me realize the importance of mastering English-to-English translation. I began to look up common diagnostic words and nursing operation words, using dictionaries or Google to find authoritative explanations. For nursing vocabulary, I would first explain them in Chinese and then translate them into simple English. However, I have also found that sometimes my explanations are too lengthy and the concise English is not fully utilized. Therefore, I revised the translated English again, if it could be solved with one word, I would never use a paragraph to explain it. And that was not enough, I would explain it to non-medical professionals again to see if they can understand. In this process, it is not only the accumulation of vocabulary and sentence patterns, but also the accumulation of professional knowledge.

In my opinion, the Nursing English learning environment was crucial. It should not be limited to classroom teaching, but should focus on nursing scenarios for learning and application, and reflect on each other through mutual communication to summarize some learning methods that are suitable for oneself. For example, I would watch medical TV dramas of my interests, such as ‘Grey’s Anatomy’ and ‘The Good Doctor’. I created a Nursing English learning environment in this way. In this way, not only can you learn some new vocabulary, but you can also learn related authentic expressions. Watching American TV shows is not just about watching Chinese subtitles, just listening to them once. I will use a three-step learning method (watch with English subtitles and note down authentic sentence structures, shadow with subtitles to learn intonation, watch again without subtitles until I can understand 90%). It may be painful at first, but as you persist, you will gradually adapt to this pattern. Moreover, in order to learn more about the communication styles of some particular medical institutions in the competition, I would consult foreign website resources, such as subscribing to videos of registered nurses in the United States on YouTube. In this way, I learned international practical nurse–patient and doctor–nurse communication skills. Finally, the practical application of Nursing English was carried out with teachers or peers for situational simulation. Through the above learning methods, we can gradually turn knowledge-based English learning into applied English learning.

With the development of China’s social economy and culture, international exchanges have become increasingly close. It is inevitable that we have to treat foreign patients in hospitals. As English is the mainstream universal language in the world, it is necessary for nursing staff to have corresponding English conversation skills. Secondly, learning Nursing English is more helpful for us to read and understand foreign nursing professional books, and lays a good foundation for future plans to go to foreign-funded hospitals or study abroad.

Along the way, I felt indifferent, tired, and did not attach importance to Nursing English at first. After discovering its charm later on, I became increasingly convinced that learning Nursing English is very interesting and challenging. Through the study of courses and the experience of competitions, the key to truly mastering a language is to organically combine the input and output of Nursing English learning, and apply what is learned.
