# Supplementary material for: Self-regulated learning strategies adopted by successful Chinese nursing students in the process of learning Nursing English
Source: PLoS One. 2024 Aug 8;19(8):e0308353. doi: 10.1371/journal.pone.0308353 (PMC11309511; doi:10.1371/journal.pone.0308353)
Supplement: S1 Data — (ZIP) [file pone.0308353.s001.zip › Data-English Version/Yang.docx]

At the beginning of entering the nursing industry, I did not make any changes in my English learning, nor did I deliberately engage in some medical English learning. Although the university offered relevant courses, I had an attitude of only participating in class. After all, for most nursing graduates, the best place for work is a less busy department in 3A hospitals. The local dialect seemed to be more important than English when communicating with patients.

This mindset didn’t change until I participated in the WorldSkills Competition in my junior year. During the competition training period, I was given the opportunity to intern at foreign-funded hospitals (United Family Healthcare Shanghai, United Family Healthcare Beijing, International Department of Renji Hospital). But the internship opportunities in foreign-funded hospitals made me realize that having excellent English skills could increase career opportunities for nursing practitioners. You don’t have to work in a foreign country to use English. But at the same time, due to the different cultural backgrounds of different native speakers, I need to learn more than simple pronunciation and grammar. My task is more about how to reasonably show empathy in English in a nurse-patient communication context and how to communicate effectively from the perspective of western mindset when patients are unwell and depressed, so as to make patients cooperate with the treatment operation.

The expression of emotions in English is closely related to tone and pronunciation. In order to speak more standard and pleasant English, I dubbed my favorite films and television shows using English dubbing apps, from reading the lines smoothly at the beginning to reproducing them with vivid emotions, then estimating the intonation of the entire sentence before dubbing, and finally listening to demonstrations to learn correct pronunciation. I have made significant progress in a short period. This progress naturally led to an increase in confidence in conversation, and speaking English became enjoyable for me.

In the practical application of Nursing English, what I need to do is not just one-way expression of the content to be expressed. Listening and expressing are equally important. Listening to potential meanings and emotions from simple English answers is a basic skill for an excellent nurse.

There were many patients who expressed negative emotions in the competition cases. At first, I would subconsciously say, ‘Don’t worry’, which is a taboo in clinical communication because it is an answer that denies the patient’s feelings. It’s just like a nurse say ‘It’s not painful’ to a cancer patient when they experience cancer pain. Similarly, ‘I understand’ is also an unreasonable answer, because it is always the patients themselves who experience pain. We are their doctors and nurses rather than patients with the same diseases. We can provide more professional answers, such as medical advice, treatment precedents, and examples of patients recovering from the same illness. This is more likely to bring hope to patients, rather than fake sympathy. How to express these contents fluently in English has become a compulsory task for me.

The participation of Nursing English teachers and foreign teachers during the training has also played an indispensable role in my progress. Only true Nursing English learners can help me shake off the expression of Chinglish. The authenticity of English is often reflected in details, such as ‘incredible’ has stronger positive emotions than ‘good or great’. The teachers also emphasized that the use of ‘Could and Would’ is important to show politeness in English communication, as it can transform imperative and commanding sentences into a gentler yet firm tone. Nurses can use this method to validate with patients and proceed with the next operation after obtaining their consent. This can not only have a soothing effect on patients, but also receive more active cooperation and response from them.

In addition to the way of speaking, eliminating the ‘knowledge gap’ in communication between nurses and patients is also a very important consideration. For example, the word palpitation may be a simple and clear word for a medical student, but for the general public, they may have never heard this word before, let alone understood its meaning. Facing patients with limited medical knowledge reserves, if nurses use heart racing, it will be more understandable for everyone. The prerequisite for smooth communication between nurses and patients is to stand at the patient’s level of knowledge to help them understand our expressions and make the feedback we need. We should truly communicate from the patient's perspective. My coach and I frequently struggled to figure out how to describe disease symptoms in clear and simple English. At this point, medical TV dramas come in handy. In some case analyses in American TV dramas, the doctors’ descriptions of their patients’ conditions were natural and accurate, and there were even vivid animated demonstrations. We could completely reproduce their description and improvise in our training. We could use paper and pens to make simple drawings when we explain to patients.

After learning English skills, the rest is to overcome fear. Many people are afraid that their English expression will be judged when talking to foreigners, so they talk to them without eye contact. This not only makes the conversation boring, but also makes themselves more and more nervous as time passes by, causing their voices trembling. This kind of fear is often more evident in large-scale competitions, where the more afraid a contestant is of making mistakes, the more they want to achieve perfection. However, the key of equal conversation between nurses and patients is never on how perfect the grammar is or how rich the conversation content is, but on truly being in the same space as the other person, immersed in the conversation, being curious and eager to explore. After 2 years of practicing Nursing English, I found that my biggest change was I am more outgoing. I am not afraid of exchanges with foreigners anymore in my daily life. I can even get along well with contestants from other countries in the competition.

In the day-after-day training for nursing competitions, my former opponents and I gradually became friends. We discussed the optimization of communication methods, practiced, and reflected together. Standing on the world stage, I interacted with and learnt from nursing practitioners from other countries, and English became a tool for me to connect seamlessly with the rest of the world.
