# Supplementary material for: Self-regulated learning strategies adopted by successful Chinese nursing students in the process of learning Nursing English
Source: PLoS One. 2024 Aug 8;19(8):e0308353. doi: 10.1371/journal.pone.0308353 (PMC11309511; doi:10.1371/journal.pone.0308353)
Supplement: S1 Data — (ZIP) [file pone.0308353.s001.zip › Data/Chen.docx]

**我对护理英语的一些看法**

我接触护理英语的契机是高考填志愿的时候，当时的我对于这个专业并不是很了解，从网上了解了一点信息后，自认为英语还可以，凑个数就报上了，巧合的是它也跟我卡上了。

后来才知道，原来这是一个全由老外们组成的教师团队来教我们。心里正感觉大事不妙，还好，除了美版的护理，中文护理课我们也是有上的。

虽然学习了中文版的护理，但毕竟课时很赶，主要的课都是美方的，所以我学的中文版护理也是常常一知半解就过去了。我的难题就在于很难把两类护理融合在一块的话，毕竟老师们的进度是不一样的，涉及面有时候也有差异，我们自己的时间也非常有限。所以除了一些浅显的问题，很多的知识在我的脑海里都是两个东西，中文的和英文的好像很难碰触些火花来。什么都学，就是双方都学不精，这是我们专业同学们得出来的一个结论，即使我们比普通护理专业来说我们每周的课算是多的，但是如果同时要把两种语言的护理学明白，我还是万万不足。

第一学年听老外上课感觉还不错，但到第二学年的时候，由于时间的不够用，老师讲解的速度越来越快（两边都是这样），于是在课后我们就需要下更大的功夫去巩固，毕竟在老外这边考试是很频繁的事情。很多人也开始对自己学习护理英语有无意义这件事感到困惑，毕竟是中国人，如果不出国留学的话，那究竟为什么要学它。而对于我来说当初这个专业也是我比较后面的选择，所以我并没有考虑太多这个专业对于我的意义，这确实是一个比较少见的专业。但是我想的是既然学了，不如就好好学。

护理英语总的来说就是学习国际国家，不止是中国的一种护理模式。怎么去认识这个新朋友，对于我来说，我认为学好护理英语的第一步，就是英语一定要过关，听、说、读、写，都很重要。第一，别的不说，老师在讲什么，还是要听懂的，虽说书上会有很多内容，但是护理知识不仅限于记忆，只有真正理解了，才能更好的消化，特别是对于神经学，沟通交流学这些更加抽象的学科来说，老师一个好的例子的讲解，是非常能帮助学习的。第二，跟中文的学习一样，老师会在课中讲解一些不在书上的内容，那些就需要自己记下来，还有一些上课不明白的点得到解答后也需要记下，这样一来英语的书写也是很重要的。最后，说和读虽然并不能明显的感觉它的重要性，但是语言的表达能加强和老师之间的交流，只有自己说对到了读对了，才能听懂老师说的英文发音。所以护理英语首先要做的就是把英语水平提高。我觉得学不好护理英文的主要原因也就是这个。

第二，就是心态要对，要坚信，你学习的是有益处的。护理英语虽然是关于国际方面的护理知识，但毕竟都是护理，跟国内护理还是有些异曲同工之妙的。很多人包括我也想过，到底对一个不出国的国内护理人员来说，它到底值不值得我们去学习。不对它进行功利性的考察来看，它对于学习护理是个好事，就算在国内不大趋流行，但我相信在日益包容的世界文化中，对外互相学习是大势所趋，围绕护理学科，取其精华去其糟粕，会让护理在各方面有更好的发展，越来越多的人会关注这门学科。长久来看，护理事业的繁荣，才能让我们这些从事护理行业的人得到保障，所以就算目前感觉没用，护理英语本身也就是有价值的。

心态对了，和其他学习一样，还要耐心，尤其是在大学里，其他学科都是期末一考就完了，只要最后加把劲就行了。不一样的是护理英语需要时常的巩固，毕竟不是母语，很多内容都是联系的，如果早早的忘了堆到最后一大考，估计是没人能学好的，外国老师的平时分占了很大的比例，所以在他们的教学体系中一个单元结束就会有一次测验，督促我们常常复习，这其实是个很有效地学习模式，虽然是被动的。如果没有耐心，很快就会半途而废了，这里，打的是持久战。

最后，再怎么英语厉害的人也要提前预习课本，因为里面很多都是专业术语，是需要提前去翻译、做功课的。我的很多课本都是标了很多中文的，随着时间的推移，懂得多融会贯通了，才有可能慢慢减少翻译量，在此之前，还是要老老实实标清楚的。

因为这个专业的特殊性，我有幸参加了一次有关护理英语的比赛。果然，俗话说“书到用时方恨少”，多学一点总是好的。在准备比赛的过程中，我重新复习了护理英语，并在模拟的国际护理场景中头一次将其运用起来。这个比赛是效仿世界技能大赛，不同的是用护理技能去照顾一位外国患者，更加具有专业性。它分为5个模块，分别是评估、交流、体位、操作和宣教。参照比赛指导书和我平时上课的内容来看，国际护理除了很讲究操作的准确、严谨性，也非常强调交流与人文关怀，因此交流在其中也是占了一大模块。而我虽然知道其中的原则，可要把这个体现出来，就考验到我了，老师也花费了很大的心力纠正我。不过这也是个好事，毕竟心理板块在护理上很容易被忽视，在我医院实习过程中，我看到国内护理情况，都是一人护理很多人，所以很难在各方面都样样做到俱全，忽略了个别病人和家属的心理。这次比赛也是提醒我，护理是在服务于整个人的身心健康，它是包括心理上的。第二次，运用到它是我在医院实习的时候，上海是个国际化的大城市，虽然在疫情下，医院里还是有些老外的身影，当时我要去给一位老外量体温和询问两便情况的时候，仿佛就回到了学习护理英语时的场景。这两件事情让我记忆犹新，在我认知中，护理英语总算迎来了它的春天。

回想我的护理英语学习经历，没有学的多好，但是它无疑是一种珍贵的财富，学习是个好东西，然而学习是没有捷径的，学习护理英语也是一样。按照自己的习惯与节奏走，把每一个PPT，每一个视频，每一张练习，每一步走踏实了，自然就会觉得轻松许多。按照目前，对于护理英语这一行业，我所知道的还是少之甚少，但我还是很感谢一些知识给我带来的，不说实际会有什么益处，但至少，这个过程，让我踏入了一个不一样的领域，一个对护理多元素的一种尝试，这种体验，也不足为差。
