# Supplementary material for: Self-regulated learning strategies adopted by successful Chinese nursing students in the process of learning Nursing English
Source: PLoS One. 2024 Aug 8;19(8):e0308353. doi: 10.1371/journal.pone.0308353 (PMC11309511; doi:10.1371/journal.pone.0308353)
Supplement: S1 Data — (ZIP) [file pone.0308353.s001.zip › Data/Lin.docx]

在我印象里，还没记事时我就接触英语了，话都说不清就在唱着字母歌了，所以从小英语从来不是我学习的难题，加上一开始就是学习的“开口英语”，故而在之后的口语考试中也没有什么困难，只是好像一直都是凭藉着所谓的“语感”支撑着，一直到高中才发现“语感”无法支持考试中的大篇幅阅读题型，英语成绩开始没有那么美观了，后来找到了一位英语老师补课，他在教学过程中不是告诉我这些题怎么选，而是告诉我怎么提升自己的英语水平，提高“语感”，正是他“授人以鱼不如授人以渔”的教学方式为我日后自主学习护理英语打下了坚实的基础。

进入大学以后第一年，英语的学习可以说是进入了停滞期，虽然有必修的课程，但为了照顾层次不齐的每位同学的英语水平，所以课程内容都较为简单，以课文解读为主。但是课堂上的小组作业展示能够很好的锻炼我们，不同的汇报主题，从自我介绍到喜欢的电影主题再到剧本展演，让我们能够讲英语真正地运用起来，不过这些运用也都只是短暂的，且范围都是日常英语。但我特别喜欢看美剧，《摩登家庭》、《吸血鬼日记》、《哥谭镇》我是一集都没落下，所以除了课堂，课外我也通过各种途径断断续续的接触英语。再后来选修的时候为了方便通过拿学分，便选择了比较有信心的护理英语，这才正式地接触到了不一样的专业英语。在课堂的学习中我发现在医学中所涉及的英语单词更为复杂，记住一个新单词仿佛在记一大堆乱码，但在学习的过程中，我逐渐摸清他们的规律，利用前缀或是后缀来区分他们，比如anti-对抗、取消、抑制、解除；veno-静脉；vaso-血管等，不过单词太多，分类太广，大多数时候我还是会选择求助翻译软件。但其实护理英语很多时候并没有那么华而不实，让人难以接近，更多的是和患者的沟通，和之前学习到的日常沟通没有什么不同，只是运用场景不同。再后来偶然的机会通过学校的横幅了解到了医护英语水平考试（METS），更多的也是应试学习，通过指定教材的刷题，熟悉考试套路最终过了考试。

护理英语真正充斥我的学习生活是从2017年的寒假开始的，那时我通过了世赛健康和社会照护项目的第一次校内选拔，而寒假的第一个学习任务就是要阅读大量的英语护理类文章，习惯使用翻译软件的我着实花费了许多时间，但整个寒假的不断学习让我把渐渐地将“语感”捡了回来。之后开学的训练，除去专业的操作技能英语也是我们的训练重点，“不够地道”、“冷冰冰”、“中式英语”、“话到嘴边说不出”一个个问题全部在英语老师面前完全暴露出来，一个个问题地改，比如口头禅“well”；假情假意的“Everything will be OK”，又或是充满歧义的“Am I clear？”和又臭又长的定语从句，为了改变这些习惯，一遍又一遍的练习，训练室我们就像不断NG的演员，不断重复，直到优化，磕磕绊绊地惊险通过了上海赛的选拔。

护理英语的学习在普通临床工作中没有任何用处，在训练的休整期我去到了医院实习时可以深刻感受到这点，国内关于护理英语学习的相关教材和资料更是少之又少，我们参考的大多都是全英文的教材，又厚又重，着实提不起学习的兴趣。但在日常提升自我护理技能方面还是有较大帮助，在大赛中为了对标国外的护理技能标准时，我们会网络上的视频资源进行学习，大多数资源来源于YouTube，其中会涉及到一些专业词汇，如果没有护理英语的学习会无法听懂或是看懂。除此之外，在大赛中我们需要向患者用尽可能简单的语言去解释医学词汇，就要做到英英互译，怎么样简短的去表达也是我会遇到的问题。在赛前我会首先对疾病有一个充分的理解，然后尽量用生动的方式去解释，比如DVT（深静脉血栓）我就会用我的手比一个圈比作血管去解释，让患者能够边听边看帮助患者理解。而英英互译的基础就需要对单词能够有着充分的理解，所以在训练过程中老师们要求我们将相关疾病做一个英语的presentation，并且能够回答出老师们的提问，虽说繁琐但却有效。接下来就是语言习惯的问题了，在大赛中我们需要评估患者的病情、指导患者康复运动、安慰患者、解决一些社会问题等，由于时间限制，我们需要用简短的话来表达清楚，可能是高中语法学习太多，所以我总喜欢说一些又臭又长的定语从句，有时候还会把自己绕进去。后来我学会用一些简短的单词，比如在做一些康复运动时，我会选择“look straight”、“hold tightly”、“left right”等，患者能够快速接受并作出动作，同时记忆起来也更加简便。但也不是所有的问题都这么“简单粗暴”，在患者向我表达一些负面情绪时我们要给予正确的反映，向他展示出我们的同情，在一开始时我会用“Don't cry”、“Don't be sad”之类的无效安慰，但在之后老师的指导和学习过程中我们用“I understand”、“I’m sorry to hear that”之类的来代替，我也会用一些开放性的问题给一些需要倾诉的患者来告诉我他的需求。

这样一来好像没有什么问题需要解决了，护理英语应该是够用了吧，但其实最致命的便是语音语调。一个案例30分钟，当我结束的时候，老师们通常都睡眼惺忪，实在是熬不过我的“催眠”音调。这个着实难以改变，过于紧张、没有真实感或许是问题原因所在，之后去到临床学习稍微有了些改善。在之后的训练中我们去到了和睦家，在那里我们接触到了全过程的英语工作环境，包括英语交班、医生的沟通与患者的沟通等。在临床学习过程中我发现老师们的沟通十分自然且流畅，同时也会注意到文化差异，比如对于疼痛的忍受，在和睦家的有关护理英语的学习才是真枪实弹，平时的纸上谈兵相比着实有些暗淡无光了。

在世赛“退役”之后，我就很少使用护理英语了，更多的可能就是在医疗美剧中吐槽取乐一下罢了。但在护理英语学习过程中的收获还是能够充实我大部分的大学学习生活。在这过程中我对于护理英语的认知不仅是复杂高大上的专业词汇，在日常和患者的沟通中所运用到的语句和我们在护理过程中说的每一句都是护理英语的学习范围。护理英语应该是有温度和感情的，能够让我们了解患者，同时也能“治愈”患者。
