# Supplementary material for: Self-regulated learning strategies adopted by successful Chinese nursing students in the process of learning Nursing English
Source: PLoS One. 2024 Aug 8;19(8):e0308353. doi: 10.1371/journal.pone.0308353 (PMC11309511; doi:10.1371/journal.pone.0308353)
Supplement: S1 Data — (ZIP) [file pone.0308353.s001.zip › Data/Liu.docx]

**护理英语学习回顾**

很荣幸能够受到王老师的邀请，回顾这10几年来的英语学习生涯。我很早之前就接触了护理这门学科，在15、16岁懵懵懂懂的时候就选择了护理专业，在上海市卫校就读。当时学习的是日语强化的护理，除了第一年的基础课程有英语外，后续两年都是日语的课程。当时我就在担心会不会日语没有学好，反倒把英语的学习也带偏了，日式英语的出名度有目共睹。尽管我当时选择了日语为主的护理专业，但是我始终没有放弃对英语的学习。在图书馆里会去主动的找英语的语法书，还有英语对话的漫画，对于一些英语为主的纪录片我也颇感兴趣。不过，改变的开始还是得从高考升学的自主招生考为契机。当时需要考语数外这三门基础课程，英语也有两年没有系统的碰了，好在对于英语的热情还是不减，我对于语言的热爱一直都是在的，这次考试也鞭策我继续学好英语。靠着短时间的课外冲刺班和试卷刷题，在学业水平考试我也获得了前15%，A级的评价。这个含金量并不高的一场”应试考试”却鼓舞了当时的我，让我觉得要再次把英语捡起来。

真正的护理英语学习，开始于大学一年级，当时的教材是《新编大学英语教程》，但是到了大学还有一系列的选修课程，我在大一的时候选修了英语沙龙，是我们学校外教的课程，一个教室有100人听课，我和我的朋友一直都坐在第一排。外教会从饮食习惯，文化，艺术，电影还有节日为主题，大大的丰富了我对英语的认识。大二，我又选择了护理英语这门选修课，这门课程也会从常见的医疗词汇，疾病，日常医院场景的对话，让我的护理专业英语也有了提高。抱着试试看的心理参加了2019年的全国大学生英语竞赛，获得了二等奖。同年，我考了医护英语（METS）三级。我一直认为护理是一门助人的学科，而且这门学科需要前进则必须了解国内外的最新的调查和进展，同时有许多药物和治疗都可能是国内刚刚起步，但是国外已经比较成熟了，这也需要医学英语和护理英语。学习词汇、语法等，是硬记的成分，但是仍然需要遵循一定的科学规律。比如前缀后缀，以及主体往往就能反映出这个单词最基本的意义。例如前缀有：anti-, homo-, gastr-，后缀有：-logy，主干词有enter等，这些词就可以组合成gastroenterology, 翻译为胃肠学。我借阅了一本由上海中医药大学出版社杨明山教授编著的《医学英语术语教程》第2版，这本书介绍医学术语的相关历史，五大基本特征、关键构词法、特殊发音规律、年代学、词频学与最常用构词形表，是一本很好用的医学英语教材。护理英语或者医学英语中有许多复合词汇存在。需要把这几部分像零件一样拼接起来。在刚开始的护理英语学习过程中，我是觉得比较困难的。医学单词通常陌生，长度很长，而且难发音。记忆的时候，刚开始就属于打开书记得是什么意思，关上书本又变成新单词的状态。这样的情况是普遍存在于同学们之间的。我当时记忆会用闪卡（flash card）辅助记忆。通常是用裁剪为1/16的A4纸，正面写上医学词汇，背面写上对应的普通单词的意思。就这样进行记忆，个人认为这个方法还是很不错的，通过这个方法记住了许多难认的单词。

在护理学习的过程中，有一些内因外因会影响到学习效果，例如缺乏动力，缺乏权威学习资料。这类问题无论是青年还是已经踏入医疗工作的前辈都会遇到。学习护理英语和英语方法很相似，是特殊和一般的关系。通过反思自己的经验，护理英语的学习最重要的还是英语专业词汇的记忆和运用。除此之外，说和写也是很重要的。在国内，有英语语言的医疗环境主要还是外资医院和医院的特需门诊。但是，现在的信息很发达，通过网络的学习以及幕课，足不出户就可以学到最先进的护理英语课程，关键就在于迈出学护理英语的第一步，并且坚持下去。

学以致用。为了锻炼护理英语的实践能力和专业素养，在大学阶段，我还参加了第46届世界技能大赛“健康与社会照护”项目上海选拔赛，这个比赛被誉为技能届的“奥林匹克”，考验的是选手的英语沟通能力、照护专业技能、解决问题的能力。通过校选，经过5轮筛选，我从几百人中筛选出来，最后在指导老师和英语语言老师的指导下代表学校参赛，获得了上海市二等奖。这也是我第一次参加全英语的护理类的比赛，给我的英语护理生涯开启了篇章。在参加完这个比赛后，我加入了世赛上海市集训队的训练，通过学习英语数据库、国际医院的见习实习，让我的护理英语能力的实战经验有了进一步提高。两年后，我又和我大学里学习中美护理项目的学妹组成搭档，参加了2021国内医学院校护理技能大赛，这同样是照护英语为母语国家的语言的竞赛。获得了学校组第一名的成绩，同时获得了个人单项操作奖。

以赛促学，在备赛过程中离不开专业指导老师和语言指导老师的悉心栽培，让我更加坚定了在护理英语领域深入学习的动力和信心，我也会继续脚踏实地，相信Every step counts

总结而言，对于我个人的护理英语学习目标是阅读能力达到无障碍阅读国外护理文献；沟通水平可以在日常医疗场景和与医疗团队沟通无阻，并且在国外医学相关论坛可以自由发表言论，不让语言成为阻碍。目前我的词汇量和护理英语能力仍然在发展阶段，离目标尚有一些距离，但是我相信通过进一步的求学和知识经验的积累，相信在未来会奋力逐步击破。护理英语的学习很漫长，同时也是充满挑战和机遇的，愿我们都能坚定的用科学的英语学习方法，打牢基础，突破局限，在护理英语的台阶上更上一层楼！
