# Supplementary material for: Self-regulated learning strategies adopted by successful Chinese nursing students in the process of learning Nursing English
Source: PLoS One. 2024 Aug 8;19(8):e0308353. doi: 10.1371/journal.pone.0308353 (PMC11309511; doi:10.1371/journal.pone.0308353)
Supplement: S1 Data — (ZIP) [file pone.0308353.s001.zip › Data/Shan.docx]

我常常说自己是一个无知的人，靠着九年义务制教育的知识活到了现在。考上卫校后，周围的同学或多或少都有点本事，有来自名声响亮的上海中学附中，有手拿证书拿到手软的学霸，有琴棋书画样样精通的，我多少是有点“菜”了，以至于我也不得不加入努力的大军。卫校的课程大致分为两类：专业知识+基础知识。我学习的专业是护理，专业知识涵盖了我学习的2/3的时间，剩余的1/3是语数英这类的基础知识。基础知识可谓是不难，但还是能出现英语可以考出个位数的同学，故基础课程开始分班，我在A班。有一位同学影响我许多，称其为学霸吧，她来自响当当名校附中，英语可谓是精英水平对强者总是自然而然的崇拜和向往，开学几个月后，学校开办了护理英语的学习班，学霸报名了，我又怎能落下？王老师这时候出现在我颇为坎坷的学习路上。初学护理英语，班上作为可谓是浩浩荡荡，学习的热情高涨，仿佛不学好了都出不去这教室。你一言，我一语，这护理英语可真简单，鼻子眼睛耳朵英语多简单啊，问问患者的名字也不难，听写的无非就是患者的各种数字，一节课后，门后就偷偷跑走了几个学生。第二节上课，画风突变，词根的学习，“acid 是酸”王老师在上面讲的头头是道，学霸在底下听的津津有味，我开始一头雾水了，满脑子的不理解，当时学的是护理英语的一级，主要的词汇有200个，在不理解的时候，死记硬背就显得非常好用，毕竟从小到大都是这么背过来的。于是我开始定计划，给自己一个小目标：一天十个单词，不出所料，坚持了6天，回顾第一个单词的时候，又熟悉又陌生的感觉，课堂小测不出意料的fail了。这感觉很抓狂，明明就有在认真的背，为什么就是分数考不高呢？学习的火苗已经熄灭了不少，几节课后，我们的英语学习班已经变成了1vs10的小班了，学霸依旧熟练消化每节课的知识，我就依旧一知半解听着王老师的讲课。课越上越难，人越上越少，在讲到“hyper词根“的时候，班上的学生也就只剩4、5个人了，大家的退却也让我在逃课的边缘来回徘徊，一度准备放弃。

学校开始一年一度国际护理技能大赛的选拔了，紧跟学霸的步伐我也参与了选拔，面试的现场感受到了实力的参差，高年资的学姐侃侃而谈，落落大方，反观自己，半句话未说变先红了脸，支支吾吾，内心谋划了半天，开口却还是最简单了“I am；I am…”，理所当然，我被淘汰了，之后我也有时刻关注训练的动向，观战比赛，强者的从容，令人向往，我重新反思自己的学习进度。一知半解的原因还是其一是词汇量的不够，我的词汇量经过测试只在2000左右，对付日常的生活或许够用，但是面对崭新的护理知识远远不够。其二是努力用错了方法。现代科技的进步就是带给我们不同的资源，在网络上我可以看到无数前辈的经验，番茄学习法，艾宾斯基遗忘曲线，看原文书…每看到一个方法，我都迫不及待的在自己身上实践，因为学的太杂，根本消化不了，目标定太大，又实现不了，我一定要找到属于自己的学习方法。俗话说的好：兴趣是最好的老师，那些事情能让我感觉到快乐呢？是大起大落的电影，是激情无限的游戏，是未知的新事物，看美剧的时候对喜欢的片段进行影子跟读，因为专业选择，看的电视剧也多跟医疗相关，譬如：《实习医生格蕾》《白色巨塔》《豪斯医生》，第一个记住的单词是cirrhosis；youtube上，会有老师一边绘画一边讲解人体的各个组织结构，快乐的同时也学习到了新的词汇；港大开发的医疗闯关游戏让人在脑筋急转弯的同时不知不觉进行记忆；专业英语只是英语的一条分支，增加词汇量必不可少，实验过无数学习app，“流利说”“扇贝”“多邻国”…“百词斩”是我用下来最有效，再打开手机的一瞬间，跳出图画的单词，利用碎片时间进行记忆；踊跃参与校内校外各种的英语比赛，从名落孙山到勇争前三护理英语的课坚持去上，从一级到四级，坚持到最后的也就四五人，时间会证明一切，我的词汇量在两年内增长到了7千，也从护理英语的小白也正式入了门。

新学期开始，换了新校区，来到浦东校区的我，参加了英语角，遇到了David——带领我感受英语魅力的老师，每周一的下午大家一起感受英语的魅力，五湖四海的口音讲述着对英语的热爱，有位来自河南的老哥让我记忆犹新，他的口语说不上流利，磕磕绊绊但毫不怯场，一开口就是一段单口相声，引得班级哄然大笑，不会的单词连比带划，大家也都能听懂，他的自信带动大家学习的兴趣，跟这样的同学同班真是一件幸事，我更愿意学习了！英语是一门艺术，它让来自五湖四海的人认识它并爱上它，在这里我对学习英语的思维也进行了转变，英语不单单是26个字母组合的文字，是主谓宾的语法句式，他是人与人之间的交流，文化的碰撞，是他们让我更加勇敢自信的用英语说出自己的想法。与此同时，我在各大医院的护理见习，与患者接触让我懂得沟通的重要性，一句话的可以有千百种含义，一开始只是为了追随强者的脚步才开始学习护理英语，一步一个脚印，我也竟成了他人眼中想要追随的“强者”，学习让我不断剖析自我，让我体会到学习的快乐，从盲目的跟随到自我提高，学习总会给你答案。

世界技能大赛的选拔赛在冬去春来之际拉开了序幕，三年的时间，我的努力也让在座的老师们看到了变化，专业成绩也表现出我的实力，成功入选后，纯英的环境进行交流让我的口语进步了不少，以前的我说英语总会主谓语颠倒，语速过快，也会经常因不知道这个单词的英文而停顿，不知所措，思维是要第一个转变，要学会自我安慰，我毕竟不是外国人，总会有不认识的单词，但是不要紧张，可以找相近意思的单词，化难为简，其次，不要总是想用高级的词汇替代，通俗易懂就是最能表达中心思想的，接着我总是语速过快，两句并一句，直到我换位思考，我才发现语速快并不能代表我的英语能力，让他人能理解才是主要的。面对患者，表现一名护士专业美的同时，关怀和理解显得尤为重要，让患者感受到语言的温度。全方位的训练也扎实了我的护理基础，两位备赛伙伴的努力更激发了我学习的热情，不仅仅局限于国内的课程，辅导老师们为我们寻求国外护理课程，中西方思想的碰撞让我感受到护理独特的魅力，同时我也感受到想要获取不同的知识，学习国外的知识。

参加完比赛后，我参与了芬兰大学的游学计划，决心去国外实地感受一下，较国内的护理，北欧属于成熟的护理体系，发展较前，书本上过于生硬，而此次芬兰之行让我形于表面的护理英语有了深度，之后长达8个月的护理实习更是让我在生活中将英语和工作结合到一起，那是一对来自巴西的父子，儿子左脚有明显外伤，步行困难，他们焦急的在急诊骨科的门口等待，可是急诊的医生正在处理另一位骨折病人，疼痛所致，小伙子支撑不住了，险些倒在了地上，父亲焦急的呼喊，这一幕在人来人往的急诊大厅并不少见，语言不同，疾病难忍，让对父子汗如雨下，正准备下班的我见到后在大厅借了一辆轮椅，并向医生说明情况，在他处理骨折病人的同时，我先询问这位小伙子的主诉，这也是我的护理英语第一次在生活中运用，“您叫什么”“来自哪里”“哪里不舒服呢”“疼痛有几分”“持续了多久呢”“calm down”“take some deep breathe”…医生处理完毕后，我将获得的信息进行告知，既减少医生重复评估又缩短患者得到治疗的时间，这段经历也大大坚定了我继续深入学习护理英语的信念。

毕业之后，我踏上了工作岗位，从事于三甲医院的王牌科室，在这里，我有更多的机会将护理英语用于实践，制作英文版的健康宣教，为外籍患者服务，介绍疾病相关知识，完成从入院-治疗-出院的一系列流程，带教同学，学习是永远没有止境的，为了完成科室的科研任务，看外籍文献成了每日的必修，晦涩又冗长的词汇极具难度，十分又挑战性，这才发现专业知识的匮乏，学习的程度时远远不够的，但也更使我沉醉其中，阅读文献使我的英语在一阶段短暂的提升了不少，通常一篇文献下来，空白的位置都写满中文的翻译，因为医学词汇的专业性，在翻译时遇到了不少困难，Google的机翻使语言变得一窍不通，只能一个一个寻找词典进行翻译，再连起来进行润色，过程很艰辛，结果是好的，在不断阅读同一疾病研究方向的论文时会对一种疾病的词汇特别敏感，反复的记忆也使我增长了不少词汇量，减少了阅读时间，增长了阅读的数量。各大医院会组织峰会，珍惜每次来之不易的机会，峰会期间各界大牛阐述自己的研究成果，崇拜之情油然而生，有了向往的目标，也就更有动力继续前行下去。

平凡的人诉说着平凡的故事，如果当时的我随众选择放弃学习护理英语，也就没有了那么多可以回味的经历，一路走来，我的英语之路也时颇为坎坷，失败失败又失败，但努力和时间会告诉我答案，对于我来说，有个向往的目标会更让我有动力，而兴趣更让我有学习的激情，让我不知疲倦，沉醉其中，我很荣幸遇到善于教学的老师，带我探索语言的魅力而不是应试教育；遇到优秀的同学，他们改变自己的同时也影响到了我；遇到沉淀的自己，为自己而学习，为自己努力。
