# Supplementary material for: Self-regulated learning strategies adopted by successful Chinese nursing students in the process of learning Nursing English
Source: PLoS One. 2024 Aug 8;19(8):e0308353. doi: 10.1371/journal.pone.0308353 (PMC11309511; doi:10.1371/journal.pone.0308353)
Supplement: S1 Data — (ZIP) [file pone.0308353.s001.zip › Data/Tang.docx]

初入大学，我首先接触到的是中美护理的课程，从而认识到了护理英语的重要性。中美护理专业除了要求牢固掌握本专业的护理知识外，还要求在日后的护理工作中能够熟练地用英语口语进行交际和掌握一定的美国实操综合技能。在整个大学时期对护理英语的自主学习是我一直所坚持和完善的，而学好护理英语在大学时期为我明确了今后的目标与方向，同时也在我的职业生涯发展中起到了指引性的作用。

大学外教的课堂相比从前的高中课堂在形式上有很大的不同。首先是讲解的内容，使用幻灯片上少量的词汇与图片进行拓展代替了高中时期传统的授课模式，同学需要将听到的重点词汇补充在随堂下发的简要笔记里，这就考验了我们的专注力和理解能力。于我而言，上课有时会听得云里雾里，例如当上一个部分还没完全理解时，老师的思维转而立即进入了下一部分。其次则是大学课堂注重听说，外教不允许我们在小组讨论的环节用中文讨论，而是将课堂内容转化为全英文交流，鼓励我们张口说英语。为了充分调动我们的积极性，外教组织了课堂辩论、看影音视频、小组答题等活动。在大学英语课上可讨论的话题也具有多样化和选择性，涵盖了影视剧、中国菜系、中国历史、中美差异等，这些话题都非常贴近于我们年轻人的生活和爱好，让我们能够畅所欲言、言之有物。令人心生涟漪的是，不论同学的英语水平的高低，外教都不会当众打断他们的发言，而是耐心地倾听，直到同学碰到了困难，老师才会使用一些词汇帮助完善同学的回答。在一声声“Good job”中，我的信心倍增，更加愿意主动面对一个个新的挑战与难题，甚至是后来可以在讲台进行公开演讲。

虽然在高中我已打下了良好的英语基础，较为牢固地掌握了各种英语语法，但是由于缺乏了一定的英语交流环境，从而养成了“哑巴英语”的坏习惯，张口将脑中的中文立即转化为英文成为了我英语学习的一大障碍，因此突然面对大学全英文的课堂，还是很难一下子适应的。所幸的是，当我在学习上碰到问题的时候，可以随时询问外教，他们还热情邀请我一同餐叙，增加练习口语的机会。与老师的关系拉近了，我自然就不再那么羞涩于张口了。

刚开学的那几周，我决定奋起直追，即刻设下学习目标，一定要赶上进度。在每天下课回到宿舍后坚持自学，除了完成当天的作业还会按照学习计划执行，包括预习明天要上的课，了解课文大概意思，抓住主题。预先查阅不懂的医学术语和生词，为单词注上注释，再将单词的含义带入课本，加强理解，这样同时也对单词加深了印象。在学习完一个章节后，我会列出一个学习大纲，复习每个章节的重点并运用思维导图将所学的知识进行归纳总结。在学习医学解剖课和医学术语课的时候我推荐身边的同学用闪卡来加强记忆，首先是分门别类地按照词根和词缀排好序，然后再将相关联的词汇写在同一张卡片上，晨起吃早饭的间隙，掏出闪卡回顾一下词汇。这些都是勤能补拙的好办法，效果也是十分显著的。总而言之，大学英语课堂给了我们相当大的自主性的同时，也带来了极大的挑战。

而后我也有幸参加了2018届上海国际护理技能大赛“慢病照护与健康促进”项目，期间护理英语起到了极大的作用。此次大赛以模拟案例和任务为基础，由外教扮演标准化病人，良好的护患沟通与规范的护理操作兼备是中美护理所注重的内涵，这正是将所学转换为所用的难得契机。正式集训的时候，由护理学老师及外语教学部老师带领本小组成员一起研究了每项操作中的细节，精心设计了每一个动作，从而使操作更加正确、规范、紧凑和美观，尽量贴近于临床。中外操作中大体不变的是人文关怀，美国护理的“CICARE”理念始终贯穿在整个过程中，印在我的脑海里。它要求我们在进行任何护理操作前都需进行自我介绍，向患者做好解释并取得配合，再进行核对，非常注重患者的隐私，关心患者感受。在一开始的演练中我过于专注在写好文稿，保证文稿的完整性与正确性上，随后老师发现了问题，接下来的一席话纠正了我的观念，为我提供了明确的方向。老师指出向患者提出问题是个双向交流的过程，要抱有积极聆听的态度，而不是只顾自己表达想说的话而忽略患者的感受。接下来，我和搭档投身于一次次的反复练习中，从一开始的流程训练到对时间的严格把控，再到应对各种突发状况的演练。最后值得庆祝的是我与搭档取得了大赛第一名的好成绩。

正如护理人际沟通课所学，接收到患者的反应后要有及时的反馈，可以通过语言、眼神交流、表情、肢体动作等，或是重复患者说的话。在批判性思维课里曾提及到的superior（优先级）的概念，在比赛的过程中得到了极大的运用，针对患者的病情有序地实施护理评估和采取护理措施，这在今后的护理工作中也是受益匪浅的，本次经历也很好地锻炼了我自己的英语水平和各项能力。

随着接诊外国患者增多，需要临床护士有很强的英语沟通能力，快速准确地采集病史、掌握病情，为医护工作带来便利。护理英语在工作中如影随形，当碰到棘手的问题时，还可以查阅文献，扩大视野，解决问题。因此我希望能够通过护理英语锤炼自己，从而能够更好的与国际护理理念接轨，去做好自己的本职工作，朝着自己擅长的领域去发展。“登高必自卑，若涉远必自迩。”我坚信凡事只有循序渐进、踏踏实实才能取得成功。
