# Supplementary material for: Self-regulated learning strategies adopted by successful Chinese nursing students in the process of learning Nursing English
Source: PLoS One. 2024 Aug 8;19(8):e0308353. doi: 10.1371/journal.pone.0308353 (PMC11309511; doi:10.1371/journal.pone.0308353)
Supplement: S1 Data — (ZIP) [file pone.0308353.s001.zip › Data/Wang.docx]

**我的中美之旅**

我在中学时，高考制度还是3+1，我选择的是物理。英语成绩一直不太好，高中英语从未及格过（事实上最接近的一次是57分），高考时阴错阳差考入了全英语授课的中美班，当时感到压力很大，如果无法听懂美籍老师的上课内容，那我呆在这里还有什么意义呢？但由于性格天生比较好强，并没有就此认输而是坚定一个信念，那就是完全相信上海健康医学院给我的教育，把所有时间都花在英语学习上，或是英语口语，或是专业英语学习。一次次，一遍遍的每天重复着，从最开始到美籍教师办公室用手比划着意思，到后来逐渐可以较为流利的表达清自己的意思，作为英语基础差而又想努力提升的人其中辛苦自然不言而喻，现简短的记录如下。

首先最重要的便是心态，要始终相信自己只要努力最终是有能力做好，可以怀着一颗谦虚的心即目前虽然位置很低，比较差但一定会有峰回路转的时候，只要一分一秒努力下去，一点一滴做下去。虽然基础不佳但我始终相信我可以做好，并且不逊色于任何人，有时候甚至有点盲目自信。

诚然，在医学英语学习中困难重重，有技术性难题如医学术语英语本身较为复杂，记忆困难。或由于全部专业课都是用英语教学，避免不了要涉及专业词汇，但事实上我认为利用英语去学习一门学科可能是英语提升的一个捷径，因为学习避免不了思考，而带着思考学习一门语言常常可达到事半功倍的效果。此外，上海健康医学院的中美班课程设置非常合理，早期有Medical Term等基础术语的教学，逐渐融入专业课的教学，前期所学的基础词汇，往往会出现在后期的专业课中，使得你的学习效果更佳。同时全课程美籍教师让我感受到了什么是纯正的英语（我中学时老师的发音还带着口音，常常令我提不起兴趣），此外对于语法的教学，美籍教师虽然在意，却不过分注重，这样就营造了轻松的氛围使得我们能够比较快乐的学习。不仅如此，课堂上美籍教师的一些做法，例如回答问题不用起立，利用一些小奖品激励，永远记得你的生日等均拉近了，学生与老师之间的距离。而良好的师生关系，也从侧面推动了英语的教与学。

当然，在专科时期的同学，并不是每一个都热爱学习的。有的不仅自己不学还不允许你学，或者自己学但不允许你学。他们仿佛对于学习有着一种天生的厌恶，对于学习的人有一种与生俱来的轻蔑，且在生活中会用各种方式打击你，有语言上的：例如会说你这么牛，你怎么高考没考进本科呢；你怎么那么装呢，你这种就是装X学霸了，还以为自己是真学霸嘛；如果你一次考试考得稍微差一些，他们便会说哟，你不是很努力吗，怎么没考好呢。也有行为上的：比如故意疏远你一些，只和自己一起打游戏的朋友交往较多，或者呢打游戏到凌晨，故意开着麦克风大声喊叫，而一大早却又不去上课，可你还得拖着疲惫的身体去上课。在面对这些状况时，我们要采取一些策略，既要解决问题，也没必要发生冲突。由于发生冲突不仅会浪费辅导员和自己的时间，并且那些所谓的同学还会认为你告状而变本加厉。于是我展开了调查，询问每间寝室的休息时间，在得到一些信息后，进行筛选询问是否还有空余床位并且乐意让我晚上过来休息。后来，有一间寝室允许我晚上过去休息且很早睡，问题得到了解决。实际上，我想表达的是，人一定要学会将不良因素转化为有利因素，比如说当有一些人令你不愉快的时候你可以把负面情绪转化为努力地动力。事实上也是这样，这些事情在我考上了本科后，室友都变得体贴多了，当你休息时，他们会带上耳机并保持环境安静。英语的学习往往受多方位影响，但能够较为合适的解决外界的不良因素也是非常关键的。

对我来说较为重要的学习活动，便是我每天上完课，一个人偷偷跑去中美楼一点一滴复习当天的内容，拖着原版书学习的时候。当时感到充实极了，我偷偷打开教室的空调，偷偷在教室大屏幕上放上课件，偷偷使用粉笔与黑板，一个人复盘着一天的学习。边看边记，只到我能用粉笔大致写出今天的学习内容，我认为人是需要一些孤独的时光的，那些时光会让你成长，那些日子我能明显感觉到随着学习的深入，我对于医学术语的反应越来越快，记忆量也越来越大。而后续的参加比赛，或在一些活动中展示，对我来说则是一个输出的过程，远没有当时一个人学习时对我的英语学习帮助大。

另外就是，我与Paul，Dave等外教的关系非常好，时至今日我已经毕业四年，我们依然时常一起吃饭，谈天说地。当时在学校时，我们时常一起吃午饭，说说自己的家庭，谈谈未来自己的理想。对于自己的口语，大家不要羞于开口，觉得自己水平不行。事实上你周围的大多数中国人是听不出你口语中的错误的，只要你的发音稍微正一点，他们就觉得你厉害的不得了，哈哈。所以不必过分在意，只要能交流，能表达，我们就可以自信一些，毕竟我们还会背一些古诗词，那要是我们一开口，外教也会很懵。所以不必过分在意，而要人为的创造英语环境，使自己置身于绝佳的英语学习环境中，要学习语言，却又不止学习语言。与外教交朋友，谈生活，将语言的学习融入日常的生活中。去看美剧，听英语歌曲，想象自己是个外国人。对于周围环境的理解，要直观，比如想到单词book要直接脑海中产生一本书，而不要book翻译为中文书再想到书。对于语言记忆学习来说，想象力，尤其是有趣的想象会使你迅速进步。

我的护理英语学习，能够完成一些交流，词汇量也基本满足日常需求，虽然也考过了一些考试比如大学英语六级，全国医护英语水平四级，但实际上还是与科班严谨的英语有差距，这点在我撰写英语论文时表现得尤为明显（英语论文需要比较完善的逻辑与独特的表达），但凡事都有解决的方法，我下载一些高分SCI论文模仿他们的表达方式与句型，时间久了也有一些收获。对我专科时期的英语学习目标我已经达成，但目前在撰写英语论文方面我还有很多学习的地方。语言的学习是一个长期的，不断前进的过程，现在来说我还是处于一个学习的阶段。我的学习方法在一定程度上是有效的，比如仅仅满足日常交流的话，但对于科研探索，论文撰写依旧有很多欠缺我依然在摸索其中的方法。希望自己能通过不断思考不断改善学习策略，争取早日靠近目标。
