# Supplementary material for: Self-regulated learning strategies adopted by successful Chinese nursing students in the process of learning Nursing English
Source: PLoS One. 2024 Aug 8;19(8):e0308353. doi: 10.1371/journal.pone.0308353 (PMC11309511; doi:10.1371/journal.pone.0308353)
Supplement: S1 Data — (ZIP) [file pone.0308353.s001.zip › Data/Wu.docx]

**以赛促学，学以致用**

我真正接触英语教育是从小学五年级开始。我对这门外语起初是很感兴趣，我喜欢26个字母，我喜欢它的书写和发音，喜欢它的简洁，喜欢听英文歌，看美剧。俗话说，兴趣是最好的老师，我逐渐在课内教育和生活中培养出很好的语感以及标准的发音，这位我后期学习护理英语打下了一个很好的基础。

我是一名护理本科生，在大学继续进行常规英语的学习，直到大学二年级的下半学期我通过比赛接触了护理英语。我报名参加了“世界技能大赛-健康和社会照护项目”的校内选拔赛，因为世界技能大赛是国际赛事，因此比赛语言是英语，这是我第一次参加以英语为主的赛事。当第一轮选拔的笔试试卷放在我面前时，我惊呆了，试卷上是全英文的单项选择题，更是一堆专业医学术语，瞬间倍感压力，我连蒙带猜的完成了考试，幸运的是我通过了。为了应对接下来的实践口语交流，我开始学习积累医学词汇，背相关句型。当这所有的前期准备在初次实践应用时还是让我不知所措，仿佛你积累了许多，但无法输出或不知道如何输出。对于护理英语，我最开始内心的想法是这无非是专业医学术语加上日常英语交流。在平日里和病人交流基础上，将对话翻译为英语罢了。这样的理解导致我与病人的对话显得生硬，医学术语频出，和标准化病人的交流没有预期中的顺畅。后来我开始向学校外教和英语老师们请教，学习了护患沟通的AIDET模式，让我逐渐对护理英语感兴趣，了解原来良好的护患关系建立是有一个语言模板的。我也逐步了解英语中的therapeutic communication，懂得了哪些表达是更适宜的，哪些表达是存在歧义的，同时也学到了一些更地道的英语表达。

起初，在以往英语学习的基础上，面对病人的抱怨，我会立马回答：Don’t worry,everything will be OK！这种表达非常常见，但也许更适用在平日里对朋友说的。而在护理英语中，我认识到这类表达也许并不是最佳的回答，病人并不会因为你的一句“don’t worry”而可以不担心，与其给予无效的安慰，还不如真诚的描述你所看到和感受到的，再鼓励病人说出内心的concern。而如今，我可以这样说：Well,you look pretty low,what made you feel this way?”“Those feelings you have are totally normal”。通过比赛，我渐渐明白，护理英语并不是纯粹的护理加英语，而是以英语这门语言为载体，将护理的专业性和人文关怀融合传递出来。

当然，在护理英语的学习过程中我也遇到了很多挑战，例如护理英语专业术语多，词汇量大，听说读写难度高。在我开始焦虑记忆这些专业词汇的时候，好在我接触了“护理英语”这门必修课，通过外教对医学词汇词根、前缀、后缀的解释，我才知道原来这些医学词汇都有相应的构词规律。只要掌握每个部分的意思，即便再难再长的医学词汇，我也会通过拆分和组建来翻译该词。俗话说好记性不如烂笔头，我每天会反复默写一些难记忆的词汇以此来加深印象。会通过查阅“医学术语学图解指导”书籍，配上图片帮助记忆。有了医学词汇的构词学习，我对护理书籍的学习和案例的解析都更容易了些。

词汇的问题解决后，接连而来的是“英译英”，那就是如何用通俗易懂的语句将一个术语解释给病人听。我记得在我第一场实践操作演练的时候，有一项任务是帮助病人预防“DVT”，这是深静脉血栓的缩写。在我开始这项任务时，病人问:“what does this mean?”而当时的我的解释是这样的“DVT means deep vein thrombosis”,然而还是没有解决病人的困惑。这件事让我意识到掌握“英译英”的重要性，我开始对常见的诊断词汇和护理操作词进行查阅，使用词典或Google来查词汇的专业解释。对于词汇的解释我会先用中文解释，继而翻译成英文，但我也发现有时候的explanation过于冗长，而英语的简洁性这个特点并未发挥出来。因而我再将翻译出的英文进行修改，若其中可以用一个词解决的，绝不用一段话来解释。而这样还不够，我会再解释给非医学专业的人听，看其是否能明白。在这过程中不只是词汇和句型的积累，同时也是专业知识的积累。

在我看来，护理英语的学习环境很重要，不应该只局限课堂内的教学，应该以护理情景为中心展开学习和应用，并通过相互交流进行反思，总结出一些适用于自己的学习方法。例如我会结合自己的兴趣看一些Medical show，如《Grey’s Anatomy》、《the good doctor》、创造护理英语学习环境，不仅可以学习到一些新词汇，还可以学习到相关地道表达。而看美剧并非单纯看中文字幕，听完一遍就好了。我会采用三步法进行学习（带英文字幕看一遍并记下较地道的表达句式、带字幕跟读学习语调、无字幕再看一遍，最后做到90%听懂）。也许刚开始会很痛苦，但坚持后，会逐渐适应这种模式。另外，为了更了解比赛中的一些特殊的医疗机构场景交流，我会查阅外网资源，例如订阅Youtube上一些美国注册护士的视频，了解国际实用护患，医护沟通技巧。最后通过找老师或同伴进行情景式模拟，进行护理英语的实际应用。通过以上一些学习方法，可以渐渐的将知识型英语学习逐步转向应用型英语学习。

随着我国社会经济文化的发展，国际间的交往越来越紧密，我们难免在医疗场所接待外籍人员。而英语作为世界主流通用语言，作为护理人员，具备相应的英语对话能力十分有必要。其次，护理英语的学习更有助于我们翻阅理解国外护理专业书籍，并且对日后想去外资医院或出国进修打下了良好的基础。

一路走来，从起初对护理英语的无感、疲惫、不重视。到后来发现其中的魅力后，我越发认为护理英语的学习十分有趣和具有挑战性。通过课程的学习和比赛的历练，将护理英语学习的输入式和输出式进行有机结合，学以致用，这是真正掌握一门语言的诀窍。
