# Supplementary material for: Self-regulated learning strategies adopted by successful Chinese nursing students in the process of learning Nursing English
Source: PLoS One. 2024 Aug 8;19(8):e0308353. doi: 10.1371/journal.pone.0308353 (PMC11309511; doi:10.1371/journal.pone.0308353)
Supplement: S1 Data — (ZIP) [file pone.0308353.s001.zip › Data/Yang.docx]

踏进护理行业之初我并没有对英语学习有什么改观，也没有刻意的去进行一些医学英语的学习，虽然大学有开办相关课程但我更多是一个仅仅到堂参与的态度。毕竟对大多护理毕业生来说最好的去向也只是在三甲医院找个不太忙的科室。在与病人交流方面，本地方言比起英语来似乎更重要。

这种心态一直到大三接触了世界技能大赛才出现了改观。在比赛集训期间我获得了在外资医院（上海和睦家，北京和睦家，仁济国际部）见习的机会，让我知道了优秀的英语能力可以增加护理从业者的择业机会，并非一定在外国工作才会用得上英语。但同时的，由于不同母语者的文化背景不同，我需要学习的不再仅仅是简单的发音、语法。我的任务更多是如何在护患交流情境下用英文合理的展示同理心和共情心。在患者身体不适且情绪低落的时候如何从“西式思维”角度做出有效的交流，从而使患者配合治疗操作。

英语中情绪的表达和声调、发音有很大联系。为了让自己说出更标准好听的词句我借用了英语配音软件，给喜欢的影视片段进行配音，从最开始的读顺台词到能声情并茂的复刻出来，再到配前先估算整句话的语调方向，最后听示范，学习正确的发声。在短短的时间内我有了很大的进步，进步自然会带来交谈上的自信，说英语变成了一件享受的事情。

与其有些不同的是，在护理英语的实际运用中我要做的不只是单向的说出要表达的内容，倾听和表达一样重要，从简单的英语回答中听出潜在含义和情绪，是一个优秀英语护士的基本功。

在大赛案例中不乏抒发负面情感的患者，起初我总是会下意识说“Don’t worry”，这在临床交流是大忌，因为这是否认患者个人感受的回答，就好像癌症病人经历癌痛时护士对他说“不疼”一样离谱。同理“I understand”也是一个不合理的回答，因为经历苦痛的永远是患者自己，我们是他们的治疗者而非病友，我们可以提供的更为专业的回答，比如医疗建议、治疗先例，比如给患者举例同病症得到治疗而康复的先例，更易给病人带去生的希望，而非听起来虚假的同情。如何将这些内容用流利的英文表达出来成了我的必修课。

在训练过程中专业英语教师和外籍教师的加入也对我的进步有着不可或缺的作用。只有真正的专业英语学习者才可以帮助我断绝中式英语的表达。英语的地道与否往往体现在小细节，比如“incredible”会比“good/great”带有更强烈的积极情绪。老师们还强调“Could/Would”的使用对于英语交流时礼貌的把控很重要，可以将祈使、命令式的句子转化为更加温柔而不失坚定的语气，护士可以用这种方式和病人validate，在获得患者的同意后再进行接下去的操作。这样不仅可以对患者起到安抚作用，也会得到患者更积极的配合和回应。

除了说话方式，在护士和患者交流中“知识层差”的消除也是很非常重要的一个考察点。比如“[palpitation](javascript:;)”这个单词对一个医学生来说是一个简单而清晰的理解，但对于普罗大众来说可能从来都没有见过这个单词，更别说了解它的含义了。面对医疗知识储备较少的患者，如果护士使用“heart racing”那便是一个大家都能理解的说法了。想要护患交流顺利的前提便是要站在患者的知识水平去帮助他们理解我们的表达从而做出我们需要的反馈。真正做到从患者的角度出发进行交流。训练期间我常常和教练为了研究一个病症如何用简单清晰的英文描述出来而苦恼的抓耳挠腮。这时医疗电视剧就派上用场了。有些case分析类美剧中医生对患者病情的描述自然又准确，甚至还有生动的动画演示，我们完全可以复刻其中的描述方式，随机应变，并在和患者解释的过程中用白纸和笔进行简单的涂画助解。

学习完英语技巧，剩下的便是战胜恐惧了。很多人在和外国人说话的时候害怕自己的英语表达会被judge，眼神躲闪的去进行对话，不仅让聊天边的索然无味，也让自己在时间一分一秒过去的过程中越来越紧张导致声音都开始发抖。这种恐惧情绪在大型竞赛时往往更加明显，选手越是恐惧出错就越想做到完美，但护患平等交谈的重点从来不是语法有多完美、也不是谈话内容多有营养，而是真正的和对方处于同一个空间，沉浸在交谈中，有好奇心和探知欲。在进行2年的医护英语练习后我发现自己最大的转变就是性格上的逐渐开朗，在生活中遇到外籍交流的机会也毫不怯场，甚至在比赛侯赛区也和别的国家的选手打成一片。

在日复一日的训练中我也和昔日的对手渐渐变成了战友，结交了深厚的友谊。我们一起讨论优化交流方式，实操演练并进行复盘。站在世界舞台上的我和来自各个国家的从业者进行交流学习，英语成为了一个帮助我和更宽广的世界无缝连接的工具。
