# Supplementary material for: Self-regulated learning strategies adopted by successful Chinese nursing students in the process of learning Nursing English
Source: PLoS One. 2024 Aug 8;19(8):e0308353. doi: 10.1371/journal.pone.0308353 (PMC11309511; doi:10.1371/journal.pone.0308353)
Supplement: S1 Data — (ZIP) [file pone.0308353.s001.zip › Data-English Version/Zhu.doc]

My first contact with Nursing English is my third academic year of nursing program study, which is the last year before clinical practice. The time was quite tight. Compared with the twelve years of full-time English study in primary school, junior high school and health school, it can only be said that it was accumulation of Nursing English vocabulary in a short period of time, shaping a rough outline of foreign-related nursing. But it had a great influence on my later study and career.

The classes in that year included 13 clinical topics: registration, admission, reception, vital signs measurement, drug administration, skin test, injection, specimen collection, preoperative and postoperative care, infection prevention, patient safety, diet education, discharge guidance, covering most departments. Each topic also provides a scene dialogue, background knowledge of related diseases, word roots and writing guidance. Even though I always have excellent performance in English, I still need to conquer these ‘friends’ who meet for the first time with interest. I have to study hard, firstly facing these highly specialized words in Nursing English.

In the first chapter of Admission, challenging words such as glomerulus and glomerulonephritis appeared. On the textbook, each new word was automatically label with a red background and marked the phonetic symbols and annotations in the word box at the end of the text. But in order to read the article smoothly and translate it more smoothly, I marked the phonetic symbols and the corresponding Chinese translation in the margins of the article. Seeing it once more reinforces my memorization. In addition, I framed some nursing-specific phrases and frequently-used dialogues within the text’ s sample dialogue sections with colored pencils. For example, is the consultant available today? Pay the registration fee. Acute upper respiratory infection, and so on. Combining with my current clinical work, looking back at the word consultant, the meanings given by the Eudic are consultant, senior consulting doctor, and consulting doctor. The explanation given by the English teacher at that time was that it was specialist clinic. I am thinking about the usage rate of using a consultant for registration in actual work and life, regardless of whether it is a regular consultant or outpatient specialist consultant. Among the patients I have met so far, they usually directly tell the name of the designated doctor, ‘Is Dr. XXX available today? Or which department should I make an appointment for? Is ENT Dr available today?’ I also checked the relevant translations, and it seems that specialist consultant is more appropriate. At my workplace, we treat consultation as a service. For example: OP Consultation new 门诊咨询新患者/ OP Consultation follow up 门诊咨询复诊患者/ OP physiotherapy new 门诊理疗新患者/ OP physiotherapy follow up 门诊理疗复诊患者. As can be seen, there is still a slight difference between knowledge learnt from textbooks and its practical applications.

As mentioned above, new words cannot be separated from word roots. What impressed me the most was – itis which means inflammation. This can be considered a frequent visitor in the study of various diseases later. Another example is ache which means pain, such as in headache, stomachache and so on. I only started to know word roots from the year of learning Nursing English. I can't help feeling that if I had known word roots earlier, learning English would have been easier. In the last year, we finished the Nursing English course. I was informed to participate in the International Nursing Skills Competition. I remember at that time we selected one outstanding nursing student in nursing student and one outstanding student in English from each class to participate in the preliminary competition. The preliminary competition is still mainly based on vocabulary assessment. Through strengthening vocabulary memorization, a top student from another class won the competition. We formed a new team to represent the school's nursing student group in the next round of competition. There is still a considerable amount of preparation time before the second round of the competition. The organizers organized a training session, which roughly introduced the format of the semi-final competition-the communication and operation between nurses and patients based on different nursing operations. To be honest, the training on that day was like listening to gobbledygook. I felt a lot of pressure when I watched practical exercises from a distance, and listened to tens of minutes of all English conversations. Thanks for the training organized by my own school later, and with visible written materials. I gradually understood and my memorization was deepened. Finally, I implemented all these into oral expression. During that period, I gradually increased my understanding and communication with my teammate, and finally managed to make some achievements.
 Looking back, at that time, I relied heavily on written materials to learn English. In a non-English environment, my listening ability was not adequate to fully understand the speakers’ key points. Perhaps I could understand if the speakers’ pace were not too fast (such as compared to the speed of VOA or media broadcasts in other English-speaking countries), and their language was not overly colloquial or local. The other person spoke slowly, and I listened and understood slowly. This is actually much more difficult than directly comparing with accurate textual materials. To put it bluntly, this is likely a common problem for most Chinese English learners. It is often easy to improve this when you are in an all-English environment, where you can significantly improve your English listening and speaking abilities.

After graduating from Shanghai Health School, I started my study in New Zealand because of this competition. I was determined to strengthen my English skills, which would be of qualitative help to my future career. Although my program is not closely related with nursing, it still belongs to the field of healthcare. As mentioned above, compared to the previous nine-year compulsory education and one year of Nursing English learning in China, two years of learning all English materials plus an all-English living and learning environment, my ability to communicate face-to-face with other people has been gradually improved. But I know that there is still a long way to go.

I remember an awkward incident during my internship at a nursing home abroad. A nurse asked me to convey to a senior citizen, ‘Have you opened your mouth?' He replied with a ‘Yes’. Later, I roughly understood that the nurse wanted to ask if the senior citizen had defecated. The conversation between them surprised me at that time. It wasn't until a long time later that I realized what the nurse was actually saying was ‘Have you opened your bowel?' When I first worked in a foreign country, my English proficiency was insufficient, and I was not familiar with the local saying wither. This absurd incident has always been remembered by me.

Later on, I officially worked as a community support worker in New Zealand, serving a group of people in the community, including teachers from the UK, paraplegic young people from India, teenagers with relatively delayed intellectual development, single mothers raising twins, middle-aged and elderly people with mental illnesses who cannot take care of themselves, and so on. New Zealand is a multicultural country. The elderly from UK told me, ‘You make me the day’. I need to communicate more with the Indian men to understand their accents. I tried to learn how to avoid provoking them and communicate effectively when caring for elderly people with Alzheimer. This is an even more difficult process for English learning. The guidance from my colleagues was really important for me. I listened to their communication styles, tone, and vocabulary used with their clients.

In my fourth year in New Zealand, I came into contact with the treatment of Chiropractic, which is still a relatively unpopular medical major in China in 2018. At that time, I found this term quite difficult to pronounce. New Zealanders refer to a spinal therapist as ‘chiropractitioner’, which is also known as ‘chiropractor’ in China. It is about the conservative therapy centered on the spine for pain in the head, neck, back, waist, sacroiliac joints, and other areas. After a course of treatment, I just planned to return to China for further development. Eventually, I joined a high-end private clinic as a doctor’s assistant, and the department I worked happened to be Chiropractic Department. Life is like this, with each part closely connected. The model of private healthcare is usually appointment based, serving commercial medical insurance holders and self-paying clients, of which 50% are foreign clients. English inevitably becomes an important cornerstone of good service. The medical operating system in English and obscure department have brought me more medical vocabulary that I have not encountered before. Medical reports submitted for preauthorization applications with insurance companies and past medical issues that require high attention during claims processing must be written in English. There are also quite a few notes taken during the early stages of my work. I have been working in this position for three years now. Our department has gradually developed from two doctors and me to seven doctors and seven assistants, including two schoolmates. During my teaching process, just like when I worked in New Zealand before, I asked them to listen more to my communication with foreign clients. For example, if a scheduled customer is late, the common and straightforward question would be: When will you arrive? In another way: Hello, Mr. XXX. Dr XXX is ready for the adjustment, are you on the way? The difference between work and study is that work is a genuine cultural exchange, accompanied by natural emotional communication, rather than a simple process of translating from Chinese to English. Improving customer satisfaction, customer-centered, and providing a good medical experience are our service principles.

Looking back on my study and work these years, Nursing English has had a significant impact on my entire life trajectory. If possible, I would prefer to increase the learning time of Nursing English in the nursing program to lay a better foundation for future foreign-related medical staff.
