# Supplementary material for: Self-regulated learning strategies adopted by successful Chinese nursing students in the process of learning Nursing English
Source: PLoS One. 2024 Aug 8;19(8):e0308353. doi: 10.1371/journal.pone.0308353 (PMC11309511; doi:10.1371/journal.pone.0308353)
Supplement: S1 Data — (ZIP) [file pone.0308353.s001.zip › Data/Zhu.doc]

第一次接触护理英语为护理学习生涯的第三个学年，也就是临床实习前的最后一年。这个时间段，于现在的我回想起来时间上颇为紧张。它相比起小学、初中、卫校这十二年的全日制英语学习，短短的一年， 只能说是短期快速的积攒护理英语的词汇量，塑造一个大概的涉外护理的雏形。 但是对我之后的学习、职业生涯产生了巨大的影响。
 那一年的课中包含了 13 个临床主题：挂号、入院接待、测量生命体征、给药、皮试、注射、收集标本、术前术后护理、预防感染、患者安全、饮食宣教、出院指导并涵盖了大部分科室。 每一个主题又提供了一个场景对话、疾病背景知识、词根词汇拓展及写作指导。即使英语是我一门中上良好的科目， 在初次面对护理英语中这些专业性极强的词汇我还是需要怀揣着一份兴趣以及钻研的劲去攻克这些初次见面的“朋友”。

在第一章入院中，就出现了 glomerulus, glomerulonephritis 这些颇具挑战的词。课本上把每个生词都自动标注了红底，并在文后的生词框中标了音标与注释。可是为了读顺文章、翻译的更通畅，我还是必要的把音标和中文再次标在了文章的边上。多看到一次，便多强化了一次记忆。并且，我又另外把对话中一些护理专用词组、常用对话用彩色铅笔框了起来。比如日常会用到的： Is the consultant available today? Pay the registration fee. Acute upper respiratory infection 等等。结合到我现在的临床工作中，回过头看 consultant 这个词，欧路词典给出的义项：顾问、 高级顾问医生、会诊医生。还有当时英语老师给出的解释是专家门诊。 我在思考，实际工作生活中用 consultant 来挂号的使用率有多高，不管挂的是普通会诊医生号还是专家门诊号。就目前我接触的患者中，他们通常会直接告诉指定的医生名字 Is Dr.XXX available today?或者说要预约哪个科室: Is ENT Dr available today? 我也查询了相关翻译，专家号用 expert 来描述似乎更多。在我工作的地方，我们会将 consultation 作为一个service。例如： OP Consultation new 门诊咨询新患者/ OP Consultation follow up 门诊咨询复诊患者/ OP physiotherapy new 门诊理疗新患者/ OP physiotherapy follow up 门诊理疗复诊患者。如此可见，书本与实际应用还是有那么一点点的差别。
 前面提到了生词，那么与生词离不开的便是词根。印象最深的便是-itis 炎症一词。这在之后各疾病的学习中可谓是个常客。 又如-ache 表示疼痛， headache, stomach 等等， 我了解词根也便是从学习护理英语中这一年才开始的，不禁感慨若是早些接触，英语学习必然会更容易一些。
 到了最后一年，护理英语的课已经完结，我像临危受命般的得到通知去参加国际护理技能大赛。记得当时是在全年级中以班级为单位，挑选护理学优秀生与英语优秀生各一位组队参加初赛。 初赛仍旧是以词汇量为主的考核，通过对词汇的强化记忆，我与另一班级中的学霸出线，组成新的一只队伍代表学校的护生组参加下轮比赛。 截止第二轮比赛前还有相当长的一段准备时间，主办方将我们组织到一起进行了一次培训，大致介绍了复赛的比赛形式会以不同的护理操作为背景进行一个护患的沟通与操作。 说实话，当天的培训对我来说就是听天书，远距离的观看实操加上好几十分钟的全英文对话，压力着实不小。全靠后期自己学校组织的培训，配合肉眼直观可见的文字材料，慢慢去理解、加深记忆，再最终落实到口头表达。那段时间里和学霸队友慢慢增加默契与沟通，最终勉强取得了些许成绩。
 回忆起来，当时的我学习英语非常依赖文字材料，在非英语环境中，我的听力达不到一个能准确理解对方说话重点的水准。也许可以明白，但是对话者的语速不能过快（如：相对起 VOA 或者英语国家的媒体播报速度等来说），语句也不能过分口语化或地方色彩。对方慢慢的说，我慢慢的去听去理解，这样的难度其实远比我直接对照百分之一百准确的文字材料来说更难。 说白了，这可能是大部分中国英语学习者的通病。而往往改善这一点很容易，置身于一个全英语的大环境中，英语的听、说能力至少可以大幅提升。
 卫校毕业后，阴差阳错的因为这场比赛，我去了新西兰留学，决心加强英语这块，对我之后的职业生涯会起到质的帮助。虽与护理打了个擦边球，但仍然属于 healthcare 这一块。两年的全英文材料，全英语的生活、学习环境，正如上所说，比起之前在国内九年义务教育加一年的护理英语学习，与人面对面的交流能力在慢慢提升，但仍旧任重而道远。
 想起在国外养老院见习时的一件尴尬事：护士让我传达一位老人“Have you opened your mouth?”老人传递回 Yes 的回复。后来我大致了解到护士想问的是老人是否排便了。这当中的对话让我当时备感诧异。直至后来很久，我才反应过来护士说的其实是“Have you opened your bowel?”初在异国他乡的工作环境， 我的英语能力不足， 抑或是当地的口头方式我们在国内不曾了解，这件荒唐事让我一直记着。
 后来我在新西兰正式入职 community support worker, 服务于社区中的群体，他们中有来自英国的老师、印度的截瘫年轻人、 智力发育相对迟缓的青少年、抚育双胞胎的单亲妈妈、精神疾病导致生活不能自理的中老年人等等等等…… 新西兰是个多元文化的国家，英国老人在我结束工作时告诉我”You make me the day.”、护理印度男士的时候我需要多交流后听懂他的口音、以及护理阿兹海默症的老人，如何避免激惹她们，如何有效沟通。这对英语是个难上加难的过程，来自同事的教导成为很重要的一环，去听她们与服务对象的沟通方式，语气、词汇。
 在新西兰的第四个年头，我接触了脊骨神经医学 Chiropractic 的治疗，这对于 2018 年的中国应该还是一个很冷门的医学专业。当时我对这个词还觉得很拗口，新西兰人称呼脊医为Chiropractitioner， 即中国称之为的 chiropractor 脊骨神经治疗师。 针对于头、 颈、 背、 腰、骶髂关节等疼痛， 以脊柱为中心进行的保守型手法治疗。 一个疗程的治疗后， 我也正好打算回到国内进行发展， 最终入职了一家高端私立诊所做医生助理， 而科室恰巧就是 Chiropractic脊柱科， 人生就是如此的一环扣一环。 私立医疗的模式通常都是预约制， 服务群体为商业医疗保险持有者与纯自费客户， 其中 50%为外籍客户。 英语不可避免地成为良好服务中重要的基石。 英文医疗操作系统与冷门的科室带给我更多没有接触过的医疗词汇。 与保险公司进行预授权申请所递交的医疗报告、 理赔时需要高度注意的既往症问题都须用英语书写。 入职初期的小笔记记录的也不算少。 到如今这个岗位做了三年， 我们科室从初期我和医生两个人慢慢发展到 7 位医生 7 位助理， 其中还有两位母校的学妹们， 我在带教过程中就像之前我在新西兰工作时一样， 让她们多听我与外籍客户的交流语句、 方式。 举个例子， 若预约客户迟到了， 普通且直白的问法： When will you arrive? 换一个方式： Hello, Mr XXX. Dr XXX is ready for the adjustment, are you on the way? 工作与学习的区别就是： 工作是真实的人文交流， 附带自然情感的交流， 而不是一个中译英的简单过程。 提升客户满意度，以客户为中心， 良好的就医体验是我们的服务宗旨。
 回顾起这几年的学习、 工作， 护理英语对我的整个人生轨迹有重大的影响。 如果可以，我更希望学制中增加护理英语的学习时长， 为未来的涉外医务人员打下更好的基础。
